# Effect of PPO/PEO Ratio on the Phase Behavior of Reverse Pluronics

**DOI:** 10.3390/polym17152061

**Published:** 2025-07-28

**Authors:** Alejandro Aguilar-Ramírez, César Alexsander Machado-Cervantes, Raúl Ortega-Córdova, Víctor Vladimir Amílcar Fernández-Escamilla, Yahya Rharbi, Gabriel Landázuri-Gómez, Emma Rebeca Macías-Balleza, J. Félix Armando Soltero-Martínez

**Affiliations:** 1 Departamento de Ingeniería Química, Universidad de Guadalajara, Blvd. M. García Barragán #1421, Guadalajara 44430, Jalisco, Mexico; alejandro.aguilar3703@alumnos.udg.mx (A.A.-R.); cesar.machado9883@alumnos.udg.mx (C.A.M.-C.); raul.ortega@academicos.udg.mx (R.O.-C.); gabriel.landazuri@academicos.udg.mx (G.L.-G.); 2 Departamento de Ciencias Tecnológicas, Universidad de Guadalajara, Av. Universidad 1115, Ocotlán 47820, Jalisco, Mexico; victor.fernandez@academicos.udg.mx; 3 LRP (Laboratoire Rhéologie et Procédés), CNRS (Centre National de la Recherche Scientifique), Université Grenoble Alpes, F-38000 Grenoble, France; yahya.rharbi@univ-grenoble-alpes.fr

**Keywords:** 10R5, 17R4, 31R1, copolymers, reverse Pluronic, phase diagram

## Abstract

The specific features of the phase diagrams of aqueous Pluronic systems, and particularly those of reverse Pluronics, are critically important for their broad range of applications, notably as nanocarriers for anticancer molecules. This work aims to investigate the effect of increasing hydrophobicity, achieved by varying the PPO/PEO ratio and the molecular weight, on the phase behavior of three reverse Pluronics: 10R5 [(PPO)_8_–(PEO)_22_–(PPO)_8_], 17R4 [(PPO)_14_–(PEO)_24_–(PPO)_14_] and 31R1 [(PPO)_26_–(PEO)_7_–(PPO)_26_]. A broad set of physical measurements, including density, sound velocity, viscosity, and surface tension, was used to characterize the physical properties of the solutions. These data were complemented by additional techniques such as direct observation, dynamic light scattering, and rheological measurements. Based on the primary measurements, molar volume, apparent adiabatic compressibility, and hydration profiles were subsequently derived. Phase diagrams were constructed for each system over concentration ranges of 0.1–90 wt.% and temperatures between 6 and 70 °C, identifying distinct regions corresponding to random networks, flower-like micelles, and micellar networks. Notably, the 31R1/water system does not form flower-like micelles, whereas both the 17R4/water and 10R5/water systems display such structures, albeit in a narrow interval, that shift toward higher concentrations and temperatures with increasing PPO/PEO ratio. Altogether, the present study provides new insights into the physicochemical behavior of reverse Pluronic systems, offering a foundation for their rational design as hydrophobic nanocarriers, either as standalone entities or in conjunction with other copolymers.

## 1. Introduction

The increasing development of drugs classified as Class II or IV by the Biopharmaceutical Classification System (BCS), characterized by low aqueous solubility, has amplified the need for effective delivery carriers [1,2]. Among the most promising carriers are copolymers, especially Pluronics (or poloxamers), which are nonionic triblock copolymers with an ABA architecture. Structurally, they are represented as (PEO)x–(PPO)y–(PEO)x, where x and y denote the lengths of the hydrophilic poly(ethylene oxide) (PEO) and hydrophobic poly(propylene oxide) (PPO) blocks, respectively [3].

Poloxamer-based hydrogels have shown considerable potential in enhancing both the solubility and the controlled release of poorly water-soluble anticancer drugs. This capability stems primarily from their intricate phase diagrams, driven by the temperature-dependent hydrophobic interactions of the PPO blocks and hydrophilic interactions of the PEO segments in aqueous environments. Such systems have proven effective in solubilizing a variety of challenging drug molecules, including silymarin, clozapine, oxcarbazepine, paclitaxel, or quercetin [4,5].

In addition to conventional Pluronics, reverse Pluronics, triblock copolymers with a (PPO)y–(PEO)x–(PPO)y architecture, have also shown promising potential as nanocarriers. Their effectiveness has been demonstrated in mixed formulations with classical Pluronics [4]. They are commercially available with a range of molecular weights and architectures, defined by the relative lengths of the PPO and PEO blocks. For instance, copolymer 17R4 has an approximate molecular weight of 2700 g/mol, consisting of approximately 1700 g/mol of PPO, and approximately 40% (≈1000 g/mol) of PEO [6].

In contrast to the extensively studied conventional Pluronics [7,8,9,10,11,12,13], reverse Pluronics have received comparatively limited attention, despite their distinct structural features and potential in drug delivery [14]. Early theoretical investigations questioned whether BAB-type copolymers, with hydrophobic PPO end blocks could form stable micellar structures in aqueous media. Subsequent studies proposed that these copolymers can indeed self-assemble into micelles, where the central hydrophilic PEO block adopts a looped conformation at the surface of a PPO-rich core, forming so-called “flower-like” micelles [15,16]. This architecture, however, imposes entropic and thermodynamic constraints. The looping of the PEO midblock increases the critical micelle concentration (CMC) and may induce structural rearrangements. Thermodynamic analyses further suggest that some hydrophobic ends can escape from the core of one micelle and become incorporated into another, facilitating the formation of interconnected micellar networks. These features underline the complexity of reverse Pluronic self-assembly and highlight the need for systematic experimental investigation [17].

Several concentration–temperature phase diagrams have been reported in the literature for reverse Pluronic systems, including: 10R5/water [18,19]; 17R4/water [20,21,22]; 31R1/water [20,23]; 25R2/water [24]; 25R4/water [24]; 25R8/deuterated water [25]. Despite differences in block composition and molecular weight, these systems share a common underlying mechanism. Below the critical micellar temperature (CMT), both PEO and PPO exhibit solubility. Upon reaching the CMT and CMC, the PPO block undergoes dehydration, leading to the induction of aggregate formation. As the temperature continues to rise above the CMT, a temperature is reached at which the solution separates into two phases, one rich in water and the other rich in copolymer. This point is referred to as the cloud point (CP).

Building on both theoretical and experimental studies, it is now well established that reverse Pluronics can adopt three types of conformations: random networks [18,19,22,25], flower-like micelles [21,22,24], and micellar networks [19,24,25]. The former two are regarded as micellar structures that emerge in the semi-dilute and concentrated regimes, respectively. In contrast, the random networks are often viewed as a pre-micellization state associated with the onset of turbidity at low concentrations prior to phase separation. Table 1 summarizes the current state of knowledge, compiling key findings from previous investigations into the phase behavior of these systems.

Recent studies have focused on the use of reverse Pluronics (RP) in mixtures, particularly with direct Pluronics (DP), to improve the structural and functional properties of the resulting systems. Incorporating RP has been proven to enhance micellar stability and promote the coexistence of both micelles. More importantly, RP is reported to enhance gelation, likely due to their ability to form interconnecting structures [11,26,27,28,29,30]. This strategy has attracted considerable interest, as early findings suggest that the presence of RP significantly enhances drug solubilization compared to DP alone [4,31]. Additionally, RP have been explored in formulations with ionic liquids, aiming to design environmentally friendly, water-free nanocarriers for drug delivery [32,33,34].

Although RP are known for their roles as defoaming, wetting, and anti-caking agents due to attributes linked to their high PPO content relative to DP [35,36,37], the potential application of flower-like micelles or flower networks remains largely unexplored. This limitation is primarily due to the scarcity of available information, which hinders the establishment of relationships between the behavior of micellar phases and the PPO/PEO ratios. Moreover, phase diagrams presented by different authors for the same RP system often show substantial discrepancies, even when the copolymer originates from the same supplier and batch, likely reflecting variations in experimental conditions or characterization methods.

For instance, the phase diagram of the 10R5/water system reported by Naskar et al. [18], exhibits substantial discrepancies in the structure formation zones when compared to the phase diagram of the same system reported by Larrañeta and Isasi [19]. Both studies reach the conclusion that this system is incapable of forming flower-like micelles due to the limited length of the PEO blocks of this copolymer. However, despite the disparity in the reported concentration intervals between the two studies, both studies delineate a zone of existence for random networks that is demarcated by a temperature differential of approximately 10 °C.

An additional example can be found in the phase diagrams of the 17R4/water system reported by Zhou and Chu [21] and Huff et al. [22], which, in contrast to the previous examples, exhibit a high degree of similarity. However, the prevailing argument in both studies is that the zones of random networks, which are not regarded as micellar structures, do not necessitate delimitation. Consequently, it is imperative to report phase diagrams for these systems, in which the zones and the structures they form can be homogenized.

This study aims to expand and consolidate current knowledge on reverse Pluronics by establishing a more systematic understanding of their phase behavior. Three RP systems, 10R5, 17R4, and 31R1 were studied in aqueous solution, selected based on their distinct molecular weight and PPO/PEO ratios, which serve as a proxy for molecular hydrophobicity. The identification of concentration and temperature ranges where unimers, random networks, flower-like micelles, and micellar networks exist was carried out by measuring and analyzing density (ρ), sound velocity (U_s_), viscosity (η_s_), and surface tension (σ). The validity of these transitions was corroborated by three independent methods: visual observations, dynamic light scattering (DLS), and rheometry. Following the collection of these results, a series of calculations was performed to elucidate the molecular interactions in detail. Detailed phase diagrams were reported for each system to aid in the search for the appropriate structure and conditions for encapsulating and controlling the release of a hydrophobic substance.

## 2. Materials and Methods

### 2.1. Materials and Solution Preparation

The three triblock copolymers that were analyzed were 10R5 [(PPO)_8_–(PEO)_22_–(PPO)_8_] MW = 2000 g/mol and HLB = 12.0–18.0, 17R4 [(PPO)_14_–(PEO)_24_–(PPO)_14_] MW = 2700 g/mol and HLB = 7.0–12.0, and 31R1 [(PPO)_26_–(PEO)_7_–(PPO)_26_] MW = 3300 g/mol and HLB = 2.0–7.0. All were procured from Merck (Darmstadt, Hesse, Germany) and used as received. The copolymers have PPO/PEO ratios of 0.96, 1.54, and 9.79, respectively, with Pluronic 10R5 being the most hydrophilic and Pluronic 31R1 being the most hydrophobic.

Double-distilled and deionized water provided by Selectropura (Guadalajara, Jalisco, Mexico) was used to prepare the dilutions and then passed through a Simplicity^®^ UV (18.2 MΩ/cm) ultra purification system (18.2 MΩ/cm) from Millipore Water (Burlington, MA, USA). Samples were prepared in new 30 mL glass vials for storage of the solution. The vials of solution were vortexed and subsequently wrapped in aluminum to prevent degradation by natural and/or artificial light sources. The solutions were allowed to reach a state of equilibrium for one week at a constant temperature of 5 °C in a refrigerator, with gentle shaking and periodic agitation, as the systems are more soluble at low temperatures.

### 2.2. Visual Tests

The physical appearance and fluidity of the solutions were determined through visual tests conducted within a temperature range of 0 °C to 80 °C, with a series of images captured at 5 °C intervals. The samples were then subjected to an isothermal bath for one hour at each temperature, with meticulous attention paid to discerning any alterations in the physical characteristics of the solutions. Two polarizing plates, one in a parallel position and the other in a crossed position, were utilized to observe the static samples and under shear stress to determine if the systems exhibited birefringence.

### 2.3. Density and Sound Velocity Measurements

Density (ρ) and sound velocity (U_s_) measurements were taken with an Antor Paar DSA 5000M density meter (Graz, Styria, Austria), which was calibrated with the same brand of ultrapure water. A stepwise temperature sweep was performed for each solution from 6 to 70 °C, with measurements taken every 1 °C and stabilized to an accuracy of ±0.001 °C. The instrument has a resolution of 1 × 10^−6^ g/cm^3^ and 1 × 10^−2^ m/s for density and sound velocity, respectively. The sample was injected into the capillary using a syringe, and the capillary automatically passed through the two measuring cells, which are arranged in series: the density cell and the sound velocity cell. This setup enables both measurements to be performed simultaneously using the oscillating U-tube method. After each experiment, the capillary was washed with a 1% solution of Alconox and double-distilled water. One syringe was used per sample to avoid contamination. Two replicates were performed per experiment, and the average of these replicates was reported along with a small discernible statistical error.

Utilizing these measurements, the apparent molar volume (V_ϕ_) and the apparent adiabatic molar compressibility (Κ_ϕ_) can be calculated by applying Equations (1) and (2), respectively [38].

(1) $$V_{\phi }=\frac{M}{\rho_{s}}-\frac{10^{3}\left(\rho_{s}-\rho_{w}\right)}{m\rho_{s}\rho_{w}}$$

(2) $${Κ}_{\phi }=\frac{10^{3}\left(\beta_{s}-\beta_{w}\right)}{m\rho_{w}}+\beta_{s}V_{\phi }$$

In Equation (1), $\rho_{s}$ denotes the density of the solution in g/cm^3^, m is the molality of the solution (moles of copolymer per kilogram of water), M is the molecular weight of the copolymer, and $\rho_{w}$ is the density of water. In Equation (2), β_s_ and β_w_ represent the adiabatic compressibilities of the solution and water, respectively. These compressibilities are calculated using Equations (3) and (4) as a function of the density and sound velocity of the solution U_s,s_ and water U_s,w_, respectively.

(3) $$\beta_{s}=\frac{10^{-3}}{U_{s,s}^{2}\rho_{s}}$$

(4) $$\beta_{w}=\frac{10^{-3}}{U_{s,w}^{2}\rho_{w}}$$

Furthermore, hydration profiles can be determined from density and sound velocity data. Equation (5) was employed to ascertain the infinite dilution hydration number (n_H0_), which signifies the hydration number as the number of copolymer moles approaches zero.

(5) $$n_{H0}=\underset{\mathrm{nd}\to 0}{\lim}\frac{n_{w}}{n_{d}}\left(1-\frac{\beta_{s}}{\beta_{w}}\right)$$

where n_w_ and n_d_ represent the moles of water and solute, respectively, in the solution. The empirical fitting of the hydration number as a function of the molarity m (moles of copolymer per kilogram of water) used was proposed by Figueroa–Ochoa [39] and is described by Equation (6), where a, b, and c are fitting parameters.

(6) $$\ln n_{H}=ae^{-\left(bm\right)}+c$$

### 2.4. Tensiometry

Semi-static surface tension measurements were performed in a portable Krüss BP50 bubble tensiometer (Hamburg, Germany). The solutions were placed in a glass cell covered by a fluid recirculation section, with the inlet and outlet connected to a recirculating bath of automotive coolant to control the system’s temperature. The tensiometer is equipped with a thermocouple that facilitates the precise measurement of the actual sample temperature, thereby enabling the adjustment of the temperature of the recirculating bath. A surface age of 1500 milliseconds (ms) was established to ensure that equilibrium was attained following the perturbation when the bubbling process commenced. Surface tension measurements were performed over a 10-min period while maintaining constant parameters to ensure a steady state. To ensure the reliability of the experimental results, three replicates were performed for each experiment, and the statistical average is reported.

The results of surface tension as a function of temperature (T) and concentration (C) were used to calculate the excess amount of copolymer adsorbed at the air–water interface (Γ) in units of mol/m^2^ with Equation (7), known as the Gibbs adsorption isotherm.

(7) $$\Gamma =-\frac{1}{RT}\frac{d\sigma }{d\ln C}$$

where R is the ideal gas constant, and linear fits were employed for the derivative term dσ/d ln C. The minimum area (a_s_) occupied by a copolymer molecule was calculated from Equation (8) [40].

(8) $$a_{s}=\frac{1}{N_{A}\left(\Gamma_{\infty }\right)}$$

where N_A_ is Avogadro’s number (6.023 × 10^23^ molecules per mol) and Γ_∞_ is the maximum amount of surfactant at the air–water interface, i.e., before CMC.

### 2.5. Dynamic Light Scattering

The dynamic light scattering (DLS) measurements were conducted using a Malvern Zetasizer Nano ZS90 from Malvern Instruments (Malvern, UK). The instrument’s light source was a four mW laser with a wavelength λ of 632.8 nanometers (nm). The instrument has a size measurement range of 0.3 nm to 10 μm and features a scattered light detector positioned at a 90° angle. The instrument utilizes the cumulant method to obtain intensity distributions.

The determination of diffusion coefficients and apparent hydrodynamic radii was achieved by implementing the Stokes-Einstein Equation (9) [40,41,42].

(9) $$r_{H}^{app}=\frac{kT}{6\pi \eta_{w}D^{app}}$$

where r_H_^app^ represents the apparent hydrodynamic radius, k is the Boltzmann constant, η_w_ denotes the viscosity of water, and D^app^ is the apparent diffusion coefficient.

### 2.6. Viscosimetry

The viscosity (η_s_) of the solutions was measured using a Lovis 2000 M-ME module from Anton Paar (Graz, Styria, Austria). The Höppler ball drop viscometer serves as an auxiliary instrument to the DSA 5000M density meter. Stepwise temperature sweeps were performed from 6 to 70 °C, with measurements taken at 1 °C intervals to an accuracy of ±0.005 °C. The measurements were obtained at a constant tilt angle of 50° as no non-Newtonian behavior was observed in the rheological analysis over the entire concentration and temperature range. The instrument has a resolution of 1 × 10^−3^ s, which is used for the precise measurement of ball rolling time. Capillaries with diameters of 1.59, 1.8, and 2.5 mm were utilized in accordance with the predetermined measurement intervals established for each individual capillary. Two replicates were performed for each experiment, and an average is reported with an imperceptible statistical error.

### 2.7. Rheometry

The experiment utilized an AR-G2 rheometer from TA-Instruments (New Castle, DE, USA). The instrument is equipped with a Peltier-type temperature controller. The selection of geometries was made according to the viscosity magnitudes of each sample. Three geometries of titanium cone and plate were utilized: one with a diameter of 60 mm and an angle of 1°, one with a diameter of 60 mm and an angle of 2°, and one with a diameter of 40 mm and an angle of 2°. All geometries were utilized in conjunction with a humidification chamber to prevent the evaporation of the samples at elevated temperatures. Prior to measuring the samples, the Peltier device was used to stabilize their temperature for one minute. Each experiment was replicated twice.

- Simple shear: Shear viscosity measurements were conducted within a speed range from 1 to 500 s^−1^, with five data points obtained for each logarithmic decade.
- The linear viscoelastic zone (LVZ) was determined by measuring the elastic and viscous moduli over a relative deformation range of 0.1 to 100% at a constant frequency of 10 rad/s, with 10 data points per logarithmic decade.
- The elastic and viscous moduli were obtained from a frequency of 0.1 to 100 rad/s at a constant strain within the LVZ. A comprehensive analysis of dynamic viscosity was conducted in conjunction with the assessment of shear rate. This analysis was complemented by the determination of viscosity, which was obtained using a viscometer.
- The elastic and viscous moduli were obtained as a function of temperature. This was accomplished at a constant frequency of 10 rad/s and with the same percentage of deformation belonging to the LVZ.

## 3. Results and Discussion

### 3.1. Visual Observations

A comparative analysis of the dissolutions of these three systems reveals notable similarities in their respective features. One of the observations is that none of the samples exhibit static or shear birefringence, indicating the absence of highly complex structures. D’Errico et al. [24] conducted a study on 25R2 and 25R4 systems, concluding that the RP require a minimum molecular weight for the formation of crystalline phases when mixed with water. This observation was made solely in the 25R4–water system. The three systems examined in this study have a molecular weight lower than 25R4 (3600 g/mol). Consequently, the findings from these tests align with the conclusions previously reported by these authors.

It has been demonstrated that alterations in coloration are observed in the three systems, with variations in both concentration and temperature. These alterations are illustrated in Figure S1 of the Supplementary Materials, wherein the dots represent the temperatures at which the transition is detected, and a visual line is employed to delineate the zones. The initial transition that was observed was from transparent to cloudy coloration. In the context of the 10R5/water system, this phenomenon occurs within a temperature range of 55 to 65 °C and a concentration range of 1 to 40% by weight. In the context of the 17R4/water system, this coloration occurs within the temperature range of 40 to 50 °C, with a concentration range of 1 to 10% by weight. However, at concentrations ranging from 15% to 40% by weight, this coloration manifests at remarkably low temperatures. For the 31R1/water system, turbidity levels were observed to range from 15 to 20 °C, with concentrations ranging from 1 to 40% by weight. This turbidity is indicative of a transition preceding phase separation in most cases. This phenomenon was also observed by Huff et al. [22] in the 17R4/water system. This characteristic has been attributed to the presence of hydrophobic PPO impurities in the copolymer. Attempts have been made to resolve this issue through filtration, but these efforts have been unsuccessful. In the present study, the samples were not purified to avoid complications in determining the actual concentration of the solutions. In this sense, working with the material without prior purification not only simplifies the experimental process but also strengthens the technological transfer of the results obtained to large-scale production processes. As Zhou and Chu [21] also observed, turbidity manifests in the dilute range of this system. The researchers reported the presence of free copolymers and “anomalous hydration” due to the “heterogeneity of the block copolymer composition”. This finding indicates that the observed coloration is attributable to the formation of a random network.

A transition from a cloudy to transparent state, occurring prior to phase separation, is also observed exclusively in the semi-dilute range of the 10R5/water and 17R4/water systems. This observation points to the presence of micelles with a flower-like morphology. Furthermore, a bluish transition was observed exclusively at the 20% concentration of 17R4/water and the 40% concentration of 31R1/water. Other researchers have identified a bluish hue in aqueous solutions of DP. This coloration is attributed to the occurrence of intense light scattering within this region, which is concomitant with micellar growth [43,44]. However, this type of coloration cannot be associated with a specific type of structure; instead, it is associated with particles of approximately 30 nanometers [45]. Concurrent studies by other researchers, e.g., Patel et al. [46], have identified a similar change in proximity to the CP, occurring just before the dehydration of the PEO blocks. This finding aligns with our observations, as it corresponds to a coloration that closely resembles the phase separation.

For the three systems, at a concentration of 50%, there are no observable visual changes, and all the solutions appear as transparent and viscous liquids. This observation was also noted by Mortensen et al. [25]. The same zone, where micellar networks were detected by rheometry and SANS, exhibits the same behavior in this work, which is discussed later. The exceptions to this behavior are the pure copolymers 10R5 and 17R4, which exhibit gel behavior from 0 to 18 °C. This critical temperature is referred to as the pour point and the manufacturer reports it as 15 °C for the 10R5 copolymer and 18 °C for the 17R4 copolymer. The pour point of the 31R1 copolymer was not observed, as it is reported to be −25 °C.

The phase separation temperatures, also known as cloud points, have been ascertained for each system. These are also delineated by the two-phase separation line (2Φ) in Figure S1. Allowing the temperature to stabilize for an extended period proved instrumental in determining these points, as they were observed visually as two distinctly separated phases, with the copolymer situated in the lower portion due to its greater density compared to water. Pérez-Sánchez et al. [20] obtained cloud point values similar to those found here for the same three systems using computer simulations. The researchers conclude that at elevated PPO/PEO ratios, “the equilibrium is readily shifted toward hydrophobic core-core interactions,” which promote aggregation and reduce the cloud point. Consequently, despite the 10R5 and 17R4 copolymers exhibiting nearly equivalent PEO concentrations, the cloud points of the former are elevated due to their significantly lower PPO content.

### 3.2. Analysis of the Micellization Process Based on Density and Sound Velocity Measurements

**Figure 1:**
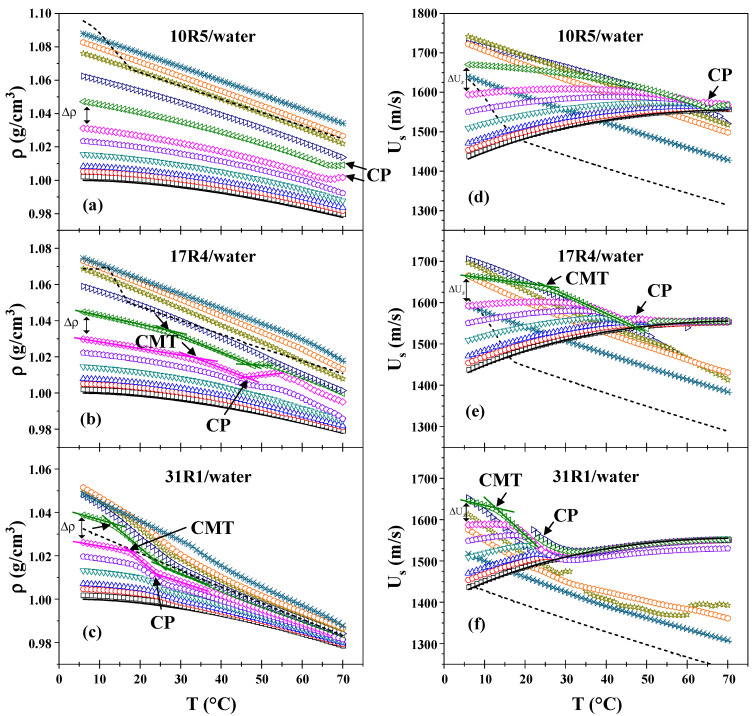
Density as a function of temperature of: (**a**) 10R5/water, (**b**) 17R4/water, and (**c**) 31R1/water; and sound velocity as a function of temperature of: (**d**) 10R5/water, (**e**) 17R4/water, and (**f**) 31R1/water. Concentrations in weight percent: 0 (─), 1 (□), 3 (○), 5 (△), 10 (▽), 15 (⬠), 20 (◇), 30 (◁), 40 (▷), 50 (☆), 60 (⎔), 80 (✳), and 100 (╍).

As illustrated in Figure 1, the density (a–c) and sound velocity (d–f) measurements are plotted as a function of temperature for the three systems. As illustrated in the graphs presented in Figure 1a–c, the density exhibited a decrease with increasing temperature and an increase with increasing concentration, by the anticipated trends. However, at a concentration of 40% by weight, the density of the three systems exhibits an almost linear behavior concerning temperature. Furthermore, the density change (Δρ) between higher concentrations is less pronounced than that between dilute and semi-dilute concentrations, a difference that becomes more evident as the PPO/PEO ratio increases. Indeed, the three pure copolymers (100%) exhibited densities nearly equivalent to those of the dilutions at concentrations of 50%, 40% and 30% for 10R5, 17R4, and 31R1, respectively. For temperatures below 18 °C, a maximum point can be observed for the pure copolymers 10R5 and 17R4. This maximum point exceeds the concentrations analyzed and is related to the pour point.

Fritz et al. [47] posit that the identification of structure formation by densimetry is of great utility because the density of hydrated PPO chains is higher than the density they have when in the form of dehydrated cores. In this study, it is demonstrated that all three systems exhibit a critical temperature at which the density undergoes a more pronounced decrease.

- In the 10R5/water system (see Figure 1a), these decays are only observed when the derivative (not shown) is analyzed. A slight increase in density is observable at 65 °C and above (at concentrations of 20 and 30 wt%), corresponding to phase separation, as confirmed through direct observation. At these concentrations, the amount of copolymer is sufficient for the instrument to begin to detect homogeneity errors in the sample.
- In the 17R4/water system (see Figure 1b), the density undergoes a precipitous decline at intermediate temperatures, ranging from 20 to 40 °C, manifesting two discernible linear zones. A comparison of the 15, 20, and 30% concentrations with the 10R5 system reveals a high degree of similarity, with both systems exhibiting a slight peak in density that rises and decays within the 45–50 °C range. This phenomenon is attributed to phase separation.
- For the 31R1/water system (see Figure 1c), the dehydration of the PPO blocks is more precipitous than for the other two systems, where two linear zones can once again be delineated between 10 and 20 °C. The phase separation of these systems was observed to occur within the temperature range of 20 to 30 °C. Above this temperature threshold, the density exhibited an almost linear behavior, with the slope of the linear regression increasing in proportion to the concentration.

Maccarini and Briganti [48] posit that a quadratic fit of the density concerning temperature can be performed up to the transition temperature, thereby enabling the evaluation of the temperature at which the maximum value the density can acquire is reached. The phase behavior of the system is determined by the temperature’s tendency to decrease or increase with increasing concentration. In the case of a structure former, the temperature rise is accompanied by a decrease in the system’s concentration. Conversely, a structure destroyer is characterized by a temperature decrease that is accompanied by an increase in the system’s concentration. The transition temperatures of all three systems under analysis decrease with concentration, indicating that all three are structure formers.

Figure 1d–f presents plots of sound velocity as a function of temperature for the three systems. At dilute concentrations and temperatures below the respective transition points, the sound velocity of the substance in question exhibits a behavior that is similar to that of water. In the case of the 10R5/water system, phase separation is discernible to the naked eye only at temperatures approaching 65 °C. In contrast, for the 17R4/water and 31R1/water systems, two linear zones are observed where the sound velocity begins to decrease at intervals consistent with those obtained by densimetry. This decline can be attributed to the formation of structures, as evidenced by Álvarez-Ramírez et al. [45]. The effective number of particles decreases as they begin to aggregate, resulting in a reduction in sound velocity.

It is noteworthy that at concentrations below 50 wt.%, all profiles converge to similar values of sound velocity after the cloud point (above 65 °C for 10R5/water, between 45 and 50 °C for 17R4/water, and between 20 and 30 °C for 31R1/water), in agreement with visual observations and densimetry. This phenomenon was previously observed in P104 copolymer by Wen and Verrall [49], who attributed this behavior to an apparent decrease in the speed of sound and the amount of aggregates with increasing concentration and temperature. This decrease is attributed to increased interactions between molecules.

As the concentration increases from 30% for the three systems, the sonic velocity demonstrates a negative slope. In a manner analogous to the densimetry results, it is evident that for concentrations ranging from 40 to 50% by weight, the ΔU_s_ undergo a reversal in trend, commencing an increase with rising concentration. This behavior approaches linearity as the temperature increases. Furthermore, it is evident that at these elevated concentrations, the particles ultimately traverse the sound velocity line of water, a phenomenon that occurs at temperatures above a certain threshold. This threshold is known to decrease in proportion to the increase in concentration. This phenomenon has been documented in earlier studies [39,45], where sound velocity values at low concentrations approach a consensus value, whereas at high concentrations, the molecular interactions are so pronounced that an apparent decrease in sound velocity is observed [45]. In the case of the 31R1/water system, higher concentrations have been shown to yield an irregular profile of the sound velocity attributed to the cloud point.

Álvarez–Ramírez et al. [45] and Figueroa–Ochoa et al. [39] investigated the phase behavior of Pluronics P103 and P104, respectively, using densimetry and sound velocity measurements. Both systems exhibited two distinct and sharp decreases in these properties as temperature increased, corresponding to the formation of spherical and cylindrical micellar structures. In contrast, the 10R5/water and 17R4/water systems exhibited a gradual decrease in density and sound velocity as temperature increased, while the 31R1/water system demonstrated a more abrupt decrease. In all cases, this behavior was followed by phase separation at higher temperatures. A comparative analysis of the three systems, both in terms of concentration and temperature, reveals that the amount of PPO plays a crucial role in lowering the transition temperatures in each system. This phenomenon can be attributed to the observation that an elevated PPO/PEO ratio results in a more pronounced dehydration of the chain, consequently leading to a more substantial decline in density and sound velocity.

**Figure 2:**
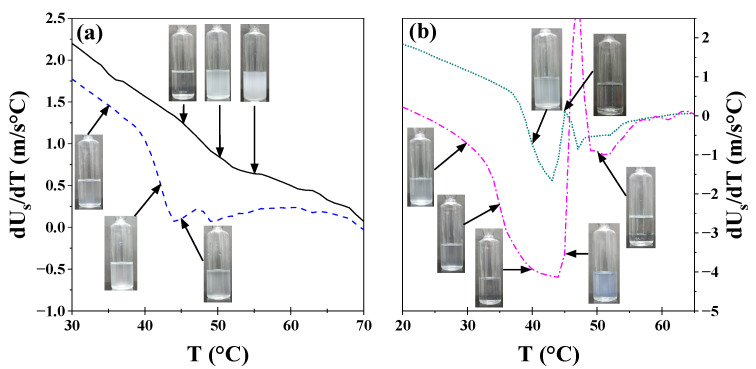
Derivative of sound velocity versus temperature of the 17R4 system from visual observations. Concentrations in weight %: (**a**) 1 (──), 5 (╌╌), (**b**) 10 (┉┉), and 20 (─∙─).

A more detailed analysis of these systems was performed by obtaining the derivative of sonic velocity and density as a function of temperature. As illustrated in Figure 2, the results for the 17R4/water system are presented. This system was selected as an exemplar among the three systems due to its transitions occurring across broader temperature intervals. The analyses for the 10R5/water and 31R1/water systems are shown in Figures S2 and S3 of the Supplementary Material, respectively.

For the 1 and 5% concentrations (see Figure 2a), a decrease in dU_S_/dT is observed between 35 and 45 °C, corresponding to the transition from transparent to cloudy, as detected in the visual tests, as shown in the insets. Between 40 and 50 °C, a second irregular change in milky color is observed, consistent with phase separation. As illustrated in Figure 2b, the same phenomenon occurs at the 10% concentration as at lower concentrations. However, the cloud point is much more visible at this concentration because the change is more abrupt, and two separate phases are observed. For the 20% concentration, two transitions are observed before phase separation, indicating the presence of a distinct structure. At these concentrations, the initial phase transition is to a visibly cloudy phase, followed by a transparent phase, and then to a bluish phase before phase separation occurs.

### 3.3. Molar Volume and Molar Adiabatic Compressibility

**Figure 3:**
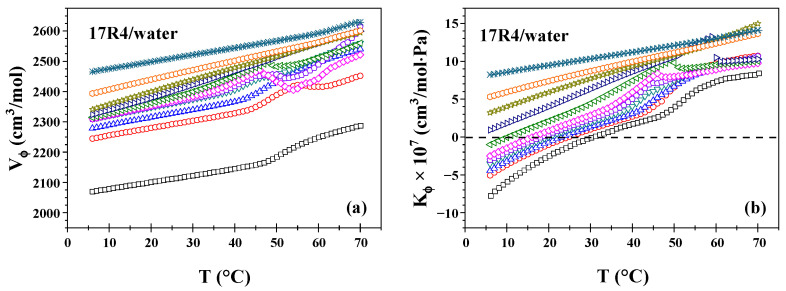
(**a**) Apparent molar volume as a function of temperature and (**b**) apparent molar adiabatic compressibility as a function of temperature of 17R4/water system. The measurements being made at the concentrations in weight percent of: 1 (□), 3 (○), 5 (△), 10 (▽), 15 (⬠), 20 (◇), 30 (◁), 40 (▷), 50 (☆), 60 (⎔), and 80 (✳).

As illustrated in Figure 3, the physicochemical calculations were obtained using density and sound velocity (Equations (1)–(4)) for the 17R4/water system. The same analyses for the systems 10R5/water and 31R1/water are illustrated in Figure S4. As illustrated in Figure 3a, the apparent molar volume (Equation (1)) demonstrates a direct correlation with temperature, exhibiting a transition in slope at temperatures associated with aggregation. This increase becomes more precipitous as the PPO/PEO ratio increases. It has been demonstrated that, at a concentration of 50%, these alterations in slope are rendered null and, concomitantly, the system exhibits monotonic behavior over the whole temperature range. Furthermore, it can be observed that, for each system, the molar volume values approach the molecular weight of each copolymer (2000, 2700, and 3300 g/mol, respectively) as the concentration and temperature increase, provided that the density of the three systems remains below 1.1 g/cm^3^.

Analogous to density, PPO blocks occupy a larger volume in their dehydrated form. In essence, the volume occupied by the copolymer molecules is greater in the micellar state compared to the monomeric state [50]. These findings are consistent with the observations reported by Wen and Verrall [49] and Álvarez–Ramírez et al. [45], who also noted a clear independence of molar volume from concentration over the approximate range of 5 to 40 wt%.

The apparent adiabatic compressibility (see Equations (2)–(4)) as a function of temperature for the 17R4/water system is displayed in Figure 3b. A more pronounced increase is observed at aggregation temperatures, except for concentrations starting at 50%, where the behavior is linear over the entire temperature range, similar to the molar volume. Furthermore, an observation of the independence of compressibility values concerning concentration reveals a range of 3 to 20% by weight. The low values of this parameter (in the order of 10–7) indicate that aggregate formation is much more influenced by temperature than by pressure. This phenomenon is anticipated in systems characterized by molecular interactions of the hydrogen bonding and Van der Waals type [49].

As posited by Armstrong et al. [51], compressibility is a highly sensitive parameter to both molecular interactions (hydrophilic and hydrophobic). It is noteworthy that in all three systems, negative values are observed over a wide range of concentrations and temperatures, despite the increasing slope towards positive values. It is important to note that Κ_ϕ_ is contingent on β_s_ and β_w_, and it assumes a negative value when β_W_ exceeds β_S_. This phenomenon can be attributed to the inherent density of the solution, which invariably exceeds that of water ($\rho_{s}$ > $\rho_{w}$). Consequently, the speed of sound exhibits significant variations in concentration and temperature, resulting in U_s,w_ > U_s,s_ due to molecular interactions [45]. This phenomenon leads to Κ_ϕ_ having negative values. This phenomenon can be rationalized by the findings of Eagland and Crowther [52], which indicate that negative values of compressibility are associated with increased hydration of the hydrophobic parts of the medium, while increasingly positive values are associated with increased dehydration of these parts of the medium. In the case of temperatures that fall below the CMT, the surfactant molecules find themselves encircled by water molecules, exhibiting a structured arrangement. As the temperature rises, the dehydration process accelerates, resulting in the observed structural breakdown. This phenomenon is particularly pronounced at the CMT, showing a heightened sensitivity to temperature changes in this region.

### 3.4. Hydration Number

**Figure 4:**
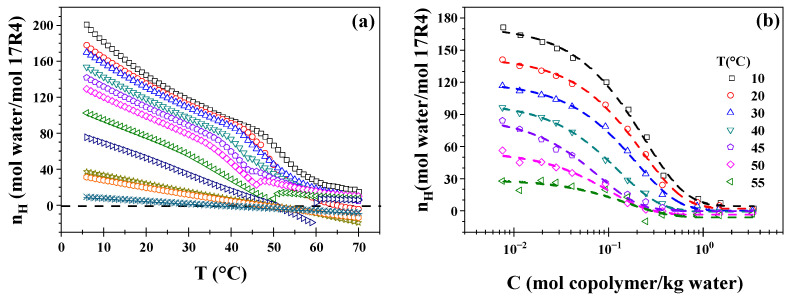
(**a**) Hydration number as a function of temperature of 17R4/water system; measurements at the concentrations in weight % of: 1 (□), 3 (○), 5 (△), 10 (▽), 15 (⬠), 20 (◇), 30 (◁), 40 (▷), 50 (☆), 60 (⎔), and 80 (✳); (**b**) Hydration number as a function of concentration of 17R4/water system.

The hydration profiles (Equation (5)) of the 17R4/water system as a function of temperature are shown in Figure 4a. As anticipated, the hydration number decreases in proportion to concentration, regardless of temperature. The same analyses for the systems 10R5/water and 31R1/water are illustrated in Figure S5a and Figure S5b, respectively. A similar behavior was previously documented by Figueroa–Ochoa et al. [39] for Pluronic P104. However, the researchers’ findings revealed a novel phenomenon: above a certain temperature threshold, the number of hydrogen-bonded molecules became independent of concentration. This transition was marked by the overlap of the concentration-dependent and -independent curves, indicating a shift in the dominant molecular mechanism. This phenomenon is not observed in the present case due to the absence of more complex structures, such as cylinders, which are commonly present in DP.

The decline in hydration number with an increase in temperature for all concentrations examined can be attributed to the inverse effect of temperature on solubility in these copolymers. The CMTs are readily discernible in this region, exhibiting a more precipitous decline, which can be attributed to the compaction of the PPO blocks. As the concentration increases, the susceptibility of the CMTs decreases. As was the case in the preceding results, it is evident that the dehydration corresponding to concentrations beginning at 50 wt.% is linear concerning temperature, exhibiting no change until before the CMT.

It is evident that as the PPO/PEO ratio increases among the three copolymers, a greater number of hydration numbers become negative. This phenomenon can be attributed to the results previously discussed in the section on sound velocity, as higher concentrations of particles are known to cross the water line due to strong molecular interactions [39].

The hydration numbers are plotted as a function of the copolymer molality in Figure 4b for the 17R4/water system and in Figure S5 of the Supplementary Materials for the 10R5/water and 31R1/water systems. These values decrease in proportion to both increasing concentration and temperature. Furthermore, an inflection point in the behavior of the profiles becomes more pronounced at a concentration of approximately 50 wt.% for the three systems. Performing the nonlinear fit (Equation (6)) proposed by Figuero–Ochoa [39] and indicated by the dotted lines in each set of experimental data allows for the determination of the hydration numbers at infinite dilution. The latter is an approximation of the number of hydrogen molecules bound to a single copolymer molecule. All measured temperatures (6–70 °C) were utilized, yielding 65 nonlinear fits for each system. The values of a, b, and c in Equation (6) were derived from the observed fits. It was determined that the sum of a and c is equivalent to the value of n_H_ at infinite dilution, designated as (n_H0_).

**Figure 5:**
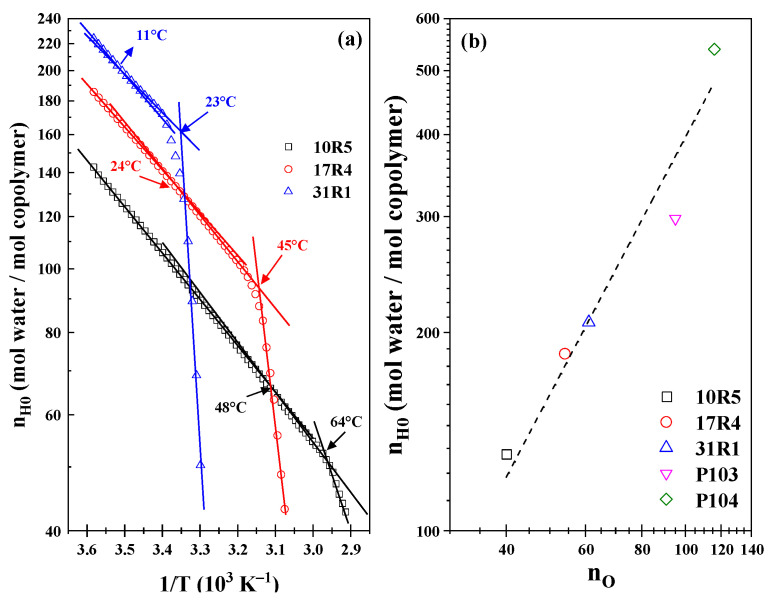
(**a**) Hydration number in infinite solution as a function of the reciprocal of the temperature; (**b**) Hydration number in infinite solution as a function of the number of oxygens in RP and DP copolymer molecules at 10 °C.

As illustrated in Figure 5a, the Arrhenius-type plot of the infinite solvation hydration number (nH0) as a function of temperature is presented. It is noteworthy that three linear zones have been identified in the three systems. The initial transition is characterized by transition points at temperatures of 48 °C, 21 °C, and 11 °C for systems 10R5, 17R4, and 31R1, respectively. However, these changes in slope must be analyzed with a proper approach because they are very weak due to their reduced ability to form structures. The second transition is associated with phase separation, where an abrupt decrease in hydration numbers is observed at temperatures of 64 °C, 45 °C, and 25 °C for the 10R5, 17R4, and 31R1 systems, respectively. Analogous to the van’t Hoff equation for a chemical reaction, the present study has obtained the dehydration energies, whose magnitudes are presented in Table 2, together with the pre-exponential factor α and the R^2^ of each linear fit.

According to the analysis by Aeberhardt et al. [53], the magnitude of the hydration numbers can be estimated by enumerating the sites at which the copolymer molecule can interact with water molecules through hydrogen bonds. According to the proposal, it is evident that the number of sites is closely related to the number of oxygen molecules (n_O_) that constitute the structure of each copolymer. The researchers obtained an excellent correlation between n_O_ and n_H0_ for different carbohydrates, which can be extrapolated to an even larger number of oxygen sites. Consequently, the following numerical values can be readily ascertained by enumerating the oxygen units: The values 40, 54, and 61 are associated with 10R5, 17R4, and 31R1, respectively. The temperature was measured at 10 °C to ensure that the systems remained below the CMT. Furthermore, the points corresponding to P103 [45] and P104 [39] were added with 95 and 116 oxygen units, respectively. As illustrated in Figure 5b, a significant correlation is evident between RP and DP, as evidenced by a logarithmic line fit, ln n_H0_ = 1.299 ln n_O_, at a temperature of 10 °C.

### 3.5. Surface Tension and Surface Area Determination

**Figure 6:**
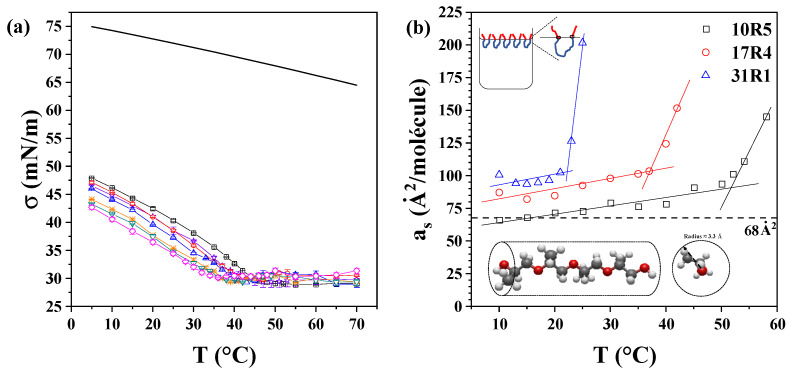
(**a**) Semi-static surface tension as a function of temperature of 17R4/water at concentrations in weight % of: 0 (──), 1 (─□─), 2 (─☆─), 3 (─○─), 5 (─△─), 7 (─✳─), 10 (─▽─), and 20 (─◇─); (**b**) Surface area as a function of temperature: 10R5/water (□),17R4/water (○), and31R1/water (△).

Figure 6a displays the semi-static surface tension of the 17R4/water system as a function of temperature. The same graph depicting the 10R5/water and 31R1/water systems is available in Figure S6 of the Supplementary Materials. It has been observed that surface tension decreases with both concentration and temperature, exhibiting a reduced slope with increasing concentration. A change in the slope of the surface tension is observed at temperatures coinciding with the transitions detected in the density and sound velocity. At temperatures above this critical point, the surface tension of the system exhibits a nearly constant value, which remains unaffected by changes in concentration. This constant surface tension is estimated to be approximately 30 mN/m for the 10R5/water and 17R4/water systems and 32 mN/m for the 31R1/water system. This phenomenon can be attributed to the compensation effect observed between the increase in the number of copolymer molecules present in the solution and the corresponding increase in the number of structures. Consequently, the activity of the copolymer in the solution remains almost constant, leading to a surface tension that exhibits minimal change with increasing concentration [54]. The irregular behavior exhibited by the 31R1 system is attributable to homogeneity errors present within the sample. These errors manifest as fluctuations in surface tension, initially diminishing to a minimum before undergoing a subsequent increase, ultimately reaching a plateau. This phenomenon is attributable to the proximity of the cloud point on one side and to the previously mentioned impurities of the sample on the other side. Similar results, attributed to impurities and broad molecular weight distributions from triblock copolymers, have been reported by Alexandridis et al. [54] and Wanka et al. [55], respectively.

The analysis of this parameter was also performed by concentration (see Figure S7 in the Supplementary Materials), where, in general, it can be observed that surface tension decreases linearly concerning the logarithm of the concentration. In addition, Wanka et al. [55] also observed a linear decrease with concentration for DP P104, P123, and P127. The Gibbs adsorption isotherm (Equations (7) and (8)) can be used to determine the area occupied by each molecule at the air-water interface, which is U-shaped to prevent contact between the PPO blocks and the water.

The structure of a 4-unit chain of propylene oxide was drawn and optimized using Avogadro software (v. 1.90.0) [56]. This was done to approximate the volume of the molecule to a cylinder. The sp3 hybridization of the carbons results in the methyl groups that characterize propylene oxide assuming four different orientations when the molecule is viewed transversely. Therefore, the radius of this cylinder is approximately equivalent to the distance from the center of the main chain to the terminus of the methyl groups. Consequently, a theoretical radius of 3.3 Å was determined, and a total area of approximately 68 Å^2^ was ascertained, considering both ends of PPO.

The experimental values of the area occupied per molecule are displayed in Figure 6b. It has been observed that the area occupied by the three copolymers displays a slightly increasing linear trend concerning temperature up to approximately the CMT values: 53 °C for 10R5, 37 °C for 17R4, and 23 °C for 31R1. The discrepancy between the calculated and observed areas can be attributed to potential entanglements at the water-air interface, which may be indicative of underlying molecular interactions. In fact, according to Goswami et al. [57], these differences are attributed to an adsorption barrier due to the change in configuration of the copolymer at the interface. It has been established that the zigzag pattern proposed for short chains is less likely to exist as the chain length increases.

These values are consistent with those reported by other authors for DP. For instance, Alexandridis et al. [54] obtained surface areas of 40 Å^2^ for P123 and 152 Å^2^ for F108, while Prasad et al. [58] obtained values of 64 Å^2^ for L62 and 146 Å^2^ for L44. In both studies, the increase in surface area was directly attributed to the content of PEO, whereas higher amounts of PPO led to a reduction in surface area, attributed to the folding of PPO chains at the interface. Conversely, in the context of reverse RP, an increase in the surface area is observed with elevated PPO content. This phenomenon is attributable to the distinct molecular configuration at the air–water interface. DP molecules adopt an inverted “U” shape, with the hydrophobic PPO block at the center, while RP molecules arrange in a “U” shape due to their hydrophobic end blocks. Naskar et al. [18], who calculated this parameter for 10R5, reached similar conclusions. It was observed that the central PPO block in DP exhibited a higher propensity for folding in comparison to the two PPO terminal blocks in RP, which were found to be hindered by steric forces, thereby limiting their folding capacity.

### 3.6. Micelle Size Measurement by Dynamic Light Scattering

**Figure 7:**
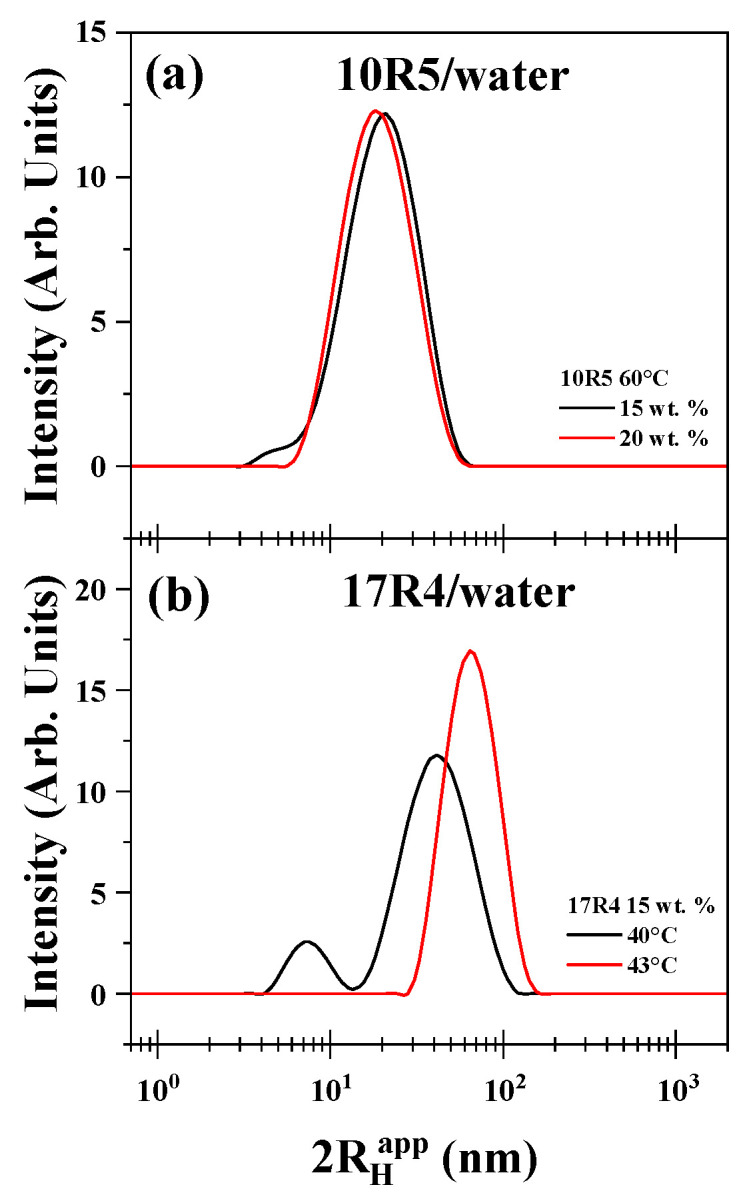
Size distributions: (**a**) at constant temperature (60°C) of the system 10R5/water at 15 (──) and 20 wt. % (──); (**b**) at constant concentration (15 wt. %) of the 17R4/water system at 40 (──) and 43°C (──).

The size distributions obtained facilitated the determination of the concentration and temperature ranges conducive to the formation of flower-like micelles. As illustrated in Figure 7a, the apparent hydrodynamic diameter of the 10R5/water system was examined at concentrations of 15% and 20% by weight at a temperature of 60 °C. The curve for 15% demonstrates a maximum of less than 10 nm, indicative of the dispersed monomer, which dissipates at the 20% concentration. A similar phenomenon occurs in the context of temperature analysis. As illustrated in Figure 7b, the diameter of the 17R4/water system was measured at temperatures of 40 °C and 43 °C, with a concentration of 15% by weight.

The 31R1/water system and concentrations below 15% of 10R5/water and 17R4/water were not suitable for measurement by this method because the polydispersity was too high and the results were not reproducible. The results obtained above indicate the presence of a transition characterized by cloudiness in the samples examined. This transition is presumed to be attributable to the presence of random networks. It is hypothesized that flower-like micelles are formed exclusively by the 10R5 and 17R4 systems in the semi-dilute regime. These systems exhibit remarkable stability with temperature, as evidenced by the absence of hysteresis in temperature sweeps that increase and decrease with the same sample (see Figure S8 in the Supplementary Materials).

The chain size of the 10R5 copolymer chain can be determined using Avogadro software, which provides an approximate extension of 14.1 nanometers. This value is consistent with the values of 14.07 ± 0.15 nm and 16.46 ± 0.51 nm for the 15 and 20% concentrations, respectively, at 60 °C.

The hydrodynamic diameter of 10R5 measured in this study at 15–20% concentration and 60 °C follows the same trend as reported in previous studies by Naskar et al. [18] and Larrañeta and Isasi [19]. In particular, Larrañeta and Isasi [19] observed values around 3 nm at temperatures below 40 °C (up to 10% concentration), followed by a marked increase to over 20 nm as the temperature exceeded this threshold, depending on concentration.

Although the present values are significantly higher than those measured at low temperatures, they are consistent with the range reported above 40 °C (between 6 and 20 nm). This agreement supports the idea that, beyond 40 °C, the hydrodynamic radius remains within the same order of magnitude across different studies, reflecting a comparable temperature-induced growth of micellar structures.

A similar outcome was observed for the 17R4 copolymer, where an approximate chain length of 19.2 nm was obtained. This value aligns with the micellar diameters of 22.24 ± 0.66 nm and 24.56 ± 1.41 nm for the 15 and 20% concentrations, respectively, at 40 °C.

This size differs from that reported for this system by Zhou and Chu [21], who reported unimers size of 1.4 nm, due to the measurement of folded unimers, because DLS assumes a hard-sphere model. The observed disparities between folded unimers and flower-like micelles serve as a confirmation of the hypothesis that unimer-unimer interactions undergo an augmentation in both temperature and concentration. However, various authors report an approximate hydrodynamic radius of 10 nanometers for the L64 copolymer, the counterpart of 17R4, in its direct form, a value that aligns with the findings of this study [59,60].

### 3.7. Rheological Characterization

**Figure 8:**
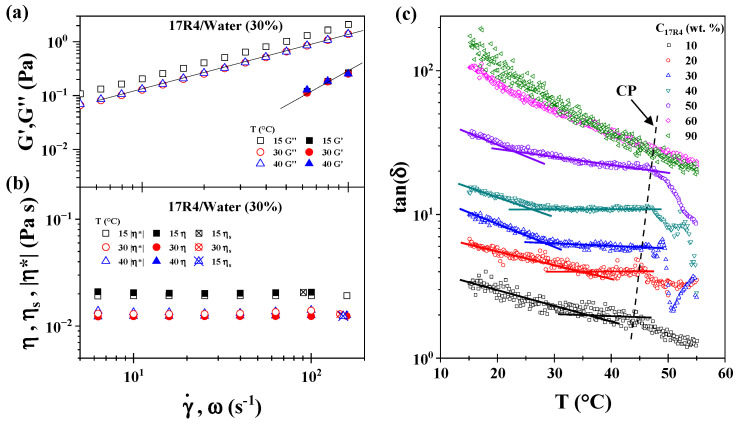
Rheometry of the 17R4/water system, (**a**) moduli G′ and G″ as a function of frequency at 40% strain, (**b**) dynamic and shear viscosity (rheometry and viscometry) as a function of frequency and shear rate, (**c**) tan(δ) as a function of temperature.

The rheological behavior of these systems is relatively simple. As illustrated in Figure 8a, the frequency sweep of the 17R4/water system at 30% concentration was conducted at various temperatures at 40% strain. It is noteworthy that all measurements fall within the linear viscoelastic zone, a notably extensive range. In general, the behavior exhibited by the three systems is characterized by a slope of 1 in the G″ modulus. However, the G′ modulus attains correct values with a slope of 2 only at high frequencies. This finding implies a predominant tendency towards viscous behavior. A lack of crossover between the moduli is evident at all observed points.

It is noteworthy that the modulus of elasticity G′ exhibits minimal variation concerning temperature and concentration. Conversely, the discrepancy between G′ and G″ increases with rising concentration and decreases with rising temperature. This phenomenon can be attributed to the predominance of viscous interactions within the systems across the entire range of parameters studied. It is noteworthy that even the values of G′ approach the lower limit of the measurement range at low frequencies. These findings demonstrate that all three systems behave as viscous liquids over the entire range studied.

As illustrated in Figure 8b, the viscosity behavior of the 17R4/water system at 30 wt.% concentration is observed at various temperatures. The filled symbols correspond to the dynamic viscosity |η*|, the empty symbols correspond to the shear viscosity determined by rheometry (η), and the crossed symbols correspond to the same viscosity but determined by a ball drop viscometer (η_s_). The complete viscometry temperature sweeps of the three systems are displayed in Figure S9 of the Supplementary Materials. It is evident that all three results are statistically equivalent and adhere to the Cox-Merz rule, a phenomenon that is not frequently observed in associative polymers [61]. The observation of this phenomenon reveals that the copolymer chains are organized in a manner that results in a high degree of similarity in their behavior under dynamic and static conditions, despite the presence of disparate deformation conditions.

As anticipated, the viscosity, presumed to be Newtonian, diminishes with an increase in temperature and escalates with an increase in concentration (illustrated in Figure S9, Supplementary Materials). However, it has been observed that within the temperature range of 30 to 40 °C, the viscosity remains relatively constant. This finding aligns with the earlier results, as aggregation occurs within this specific temperature range. This phenomenon of superposition also manifests in the G′ modulus during the frequency sweep. This phenomenon is noteworthy because, in the presence of aggregation, the rheological properties do not undergo a sudden change; instead, they remain constant. The aforementioned phenomena can be more clearly delineated in Figure 8c, which illustrates tan(δ) as a function of temperature across a range of concentrations within the 17R4/water system. This relationship is sustained throughout the interval above 1, thereby confirming that G′ is invariably higher and that the system is predominantly viscous.

Furthermore, the temperature data are displayed in regions where the decrease of this parameter remains relatively constant until just before the cloud point (dotted line). These points correspond to those obtained with the previously mentioned methods. Once more, the trend changes from the 50% concentration onwards, as the tan(δ) of the higher concentrations nearly overlap. An overlap exists between the 60% and 90% concentrations; however, this overlap is influenced by the fact that the 60% sample separates into two distinct phases. One potential explanation for the remarkably similar values of tan(δ) within this interval is the presence of crowding due to the larger structure.

The results discussed thus far have revealed peculiarities that emerge at concentrations of 50% and above. Mortensen et al. [25] observed a decline in the elastic and viscous moduli for the 25R8 system at this concentration, indicating a transition to a viscous liquid phase at elevated temperatures, accompanied by a nearly constant G′ value. Using SANS, the researchers identified the presence of micellar networks in this area. This network consists of spherical micelles that share PPO blocks from core to core, thereby establishing connections between them. This phenomenon likely underlies the absence of complex behavior observed in these networks.

Additionally, it is noted that the viscosity of these networks is Newtonian when analyzed in single shear, and that they remain transparent liquids over the entire temperature range. Consequently, our results are in complete agreement with these findings. The 10R5/water and 31R1/water systems demonstrate behavior analogous to that previously observed (see Figures S10 and S11 in the Supplementary Materials).

### 3.8. Analysis by Concentration

**Figure 9:**
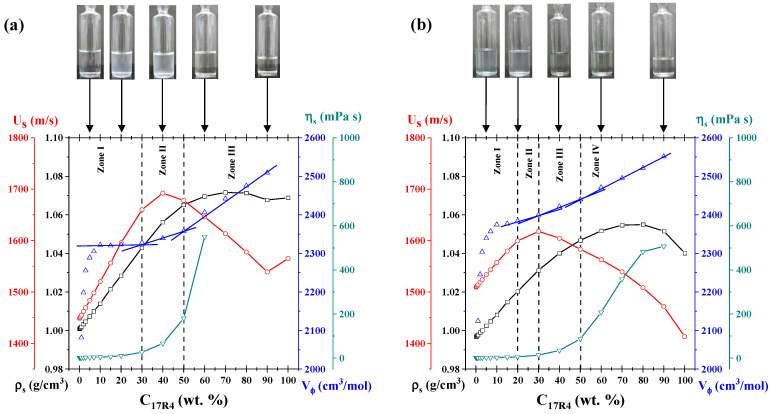
Example of analysis by concentration of the 17R4/water system: (**a**) 10°C and (**b**) 30°C. Property measurements: density (─□─), sound velocity (─○─), molar volume (─△─) and viscosity (─▽─).

To confirm the presence of other types of aggregates at elevated concentrations, plots analogous to those depicted in Figure 9 were utilized. The figure presents an illustration of the concentration analysis of density, sound velocity, viscosity, and molar volume at the 10 and 30 °C isotherms for the 17R4 copolymer. The same analyses for the 10R5/water systems at 20 °C and 40 °C, and for 31R1/water at 6 and 15 °C, are shown in Figures S12 and S13 of the Supplementary Materials.

At the 10 °C isotherm (see Figure 9a), three primary zones are identified. In zone 1, the characteristic cloudy coloration of the solutions of this copolymer is observed in the unimer zone, as previously reported by Huff et al. [20]. Furthermore, the sound velocity undergoes a linear change with concentration up to a threshold of 30%. At this point, an inflection point is reached, leading to a maximum value and subsequent decrease, which corresponds to zone II. This parameter exhibits a negative linear slope concerning concentration from 50% (zone III). This corresponds precisely to the concentration at which the samples no longer exhibit visual changes. It has been demonstrated that sonic velocity is directly proportional to the number of particles in the solution [62]. Consequently, this behavior, in accordance with the principles of temperature analysis, corroborates the presence of larger aggregates. These alterations manifest at analogous concentrations in density, viscosity, and molar volume outcomes: the augmentation in density results in a change in slope, the escalation in viscosity occurs abruptly, and the molar volume exhibits discernible linear zones. These zones are delineated by the transitions “unimers-unimers” and “micellar networks-micellar networks.”

In the 30 °C isotherm (see Figure 9b), discernible transitions are evident due to the accentuated changes in slope, which are particularly evident in the molar volume. In zone I, a cloudy solution is observed with increasing concentration below 20% by weight; this coloration corresponds to that acquired by the copolymer solutions in the unimer zone. Between 20 and 30%, the initial alterations in the slope of the four properties examined are evident; thus zone II corresponds to the emergence of random networks identified in the temperature analysis. Between 30 and 50% by weight, zone III appears, which also coincides with the temperature analysis carried out for the spherical micelles, along with the transition to transparent solutions. Up to 50% by weight, another change in the slope occurs, very similar to the previous ones, which is attributed to the formation of micellar networks (zone IV). This behavior was also observed by Mortensen et al. [25] in remarkably similar concentration ranges for the 25R8 system.

The molar volume behavior is the most notable. González–Perez et al. [63] observed analogous behavior for alkyl dimethyl benzyl ammonium chloride surfactants with varying hydrophobic chains. The researchers attributed the initial increase to the increasing volume of the micelles until an almost constant value was attained. At concentrations that exceeded a certain threshold, they detected an additional slight increase, which was attributed to the micellar transition from spheres to cylinders. For the three systems analyzed here, the initial increase, which occurs from 1 to 10% concentration (zone I of both isotherms), is likely attributable to the increasing amount of unimers. The subsequent increase is associated with a structural transition to a larger size.

### 3.9. Phase Diagrams

**Figure 10:**
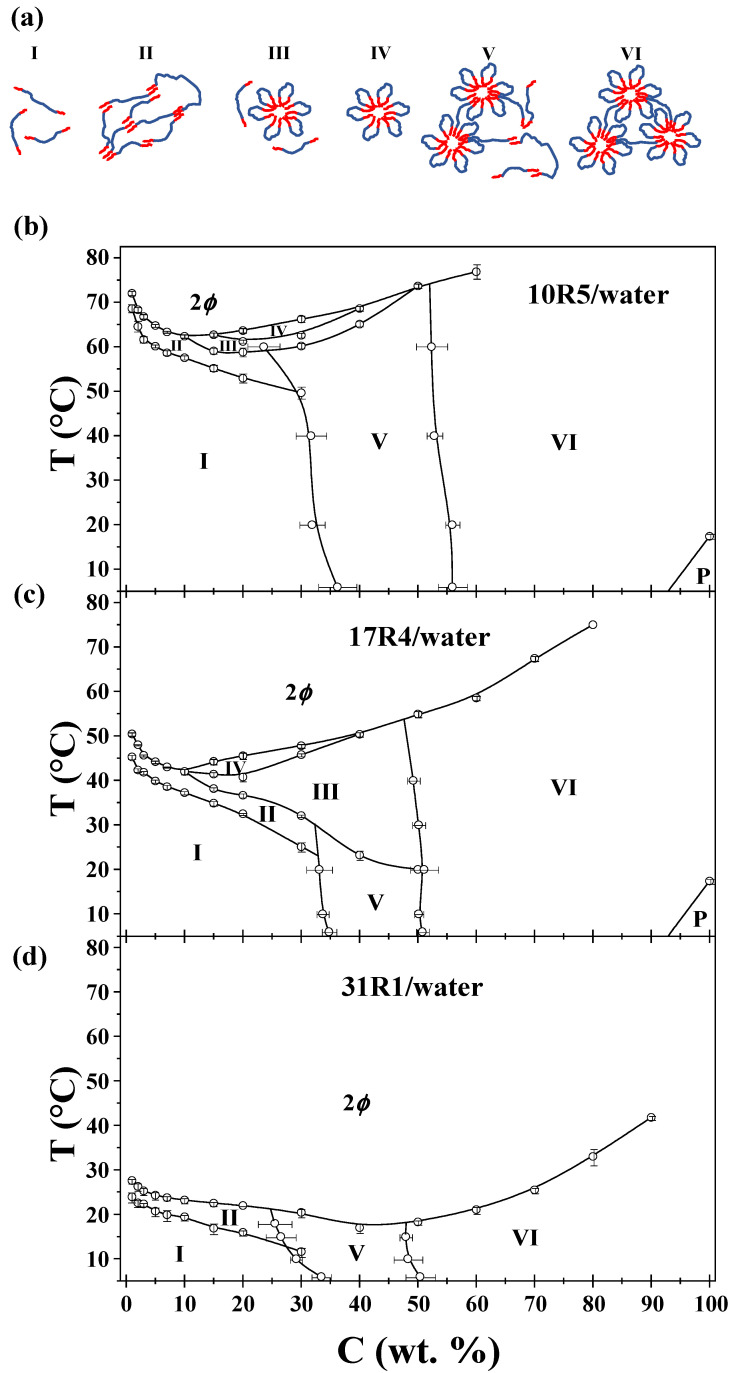
(**a**) Phase Nomenclature: PPO segments are shown in red and PEO segments in blue. Phase diagrams of the systems: (**b**) 10R5/water, (**c**) 17R4/water, and (**d**) 31R1/water.

As detailed in the preceding analysis, a total of six possible conformations were identified: unimers, random networks, flowers and unimers, flowers only, micellar networks, and unimers and micellar networks only. These are illustrated and enumerated in Figure 10, alongside the phase diagrams of the three systems. Furthermore, the phase separation is denoted by 2ϕ, and the gel behavior of the pure copolymers 10R5 and 17R4 is denoted by P. The points obtained here correspond to an average between the transition temperatures and concentrations obtained in all the methods and calculations. The standard deviation in temperature or concentration is also provided, depending on the method of analysis.

The factors considered in order to identify the phase corresponding to each of the transitions are as follows (see Figure 10a for visual representations of these factors):

I. Unimers: visually transparent with high polydispersity and monotonous physicochemical properties.
II. Random networks: visually opaque with minor changes in viscosity, density, and sound velocity.
III. Flower-like micelles and unimers: optically transparent with significant changes in viscosity.
IV. Flower-like micelles: visually similar to zone III. Zones III and IV are distinguished by DLS size distributions.
V. Micellar networks and unimers: Visually transparent and with abrupt changes in density, sound velocity, viscosity, and molar volume when analyzed by concentration.
VI. Micellar networks: Visually similar to Zone V. Zone V and VI can be distinguished by the beginning and end of the transition development, as illustrated in Figure 10.

As illustrated in Figure 10b, the phase diagram of the 10R5/water system is characterized by the presence of specific phases. This is analogous to the wedge shape reported by Naskar et al. [18] at low concentrations; however, our values differ by nearly 10 °C. This discrepancy can be attributed to the factors inherent to the copolymer manufacturer. They also note that this system only forms random networks within this specific range. Larrañeta and Isasi [19] also present a phase diagram for this system that differs significantly from the diagrams reported by Naskar and in the present study. This discrepancy is most likely because they only used DLS and visual observations as the sole characterization method. Notwithstanding, the diagram obtained in this study aligns with the one presented in the aforementioned work for the formation of micellar networks above a concentration of 50 wt%.

The phase diagram of the 17R4/water system is illustrated in Figure 10c. The CMT behavior and magnitudes of this system are in good agreement with those reported by Zhou and Chu [21] and Huff et al. [22]. In both works, the researchers identified only the flower-like micelle zone. This is because they considered random networks as not being a structure, and hence, did not strictly correspond to the CMT. Consequently, there is a discrepancy among authors regarding what is referred to as CMT for these systems. In the present study, we identify it as the temperature at which the PPO block becomes insoluble, i.e., also considering random networks. It is noteworthy that this system has the PPO/PEO ratio that favors micellization in RP. The phase diagram in this study bears a striking resemblance to that reported by Huff et al. [22], corroborating that while impurities can exert an influence on the kinetics of the aggregation process, as evidenced by surface tension data, they do not exert a significant impact on the CMT values.

The phase diagram of the 31R1/water system is illustrated in Figure 10d. In this system, the absence of flower-like micelles is attributable to the limited length of the central PEO block, which hinders the formation of a stable micellar structure. This diagram bears a striking resemblance to the phase diagram of the 25R8-deionized water system by Mortensen et al. [25], as both studies concluded that only random networks and micellar networks exist.

A comprehensive analysis of the three plots reveals several notable factors. It is noteworthy that the micellar networks are observed at approximately the same 50% concentration. Furthermore, an evident outcome of elevating the PPO/PEO ratio is manifest. This is due to the fact that 10R5 signifies hydrophilic extremity, while 31R1 denotes hydrophobic extremity. In both cases, the formation of flower-like micelles is disfavored: in 10R5 due to its short PPO arms and 31R1 due to its short PEO center. It is also noteworthy that the CMTs and CPs are inversely proportional to the PPO/PEO ratio, since these transitions occur at lower temperatures as the latter increases. Finally, it is interesting to analyze the phase diagrams of copolymers 10R5 and 17R4 independently, given that both contain nearly equivalent numbers of PEO units (22 and 24, respectively). This finding underscores the pronounced impact of augmenting PPO arms on the process of flower formation.

The three copolymers exhibit a similar structural transition in zone II, which corresponds to the wedge-shaped monomer zone. The concentrations and temperatures of this transition (zone I to zone II) were used to calculate the changes in state properties during the PPO dehydration process using Equation (10), in which x represents the mole fraction of dehydration and T the temperature, and in which ΔH represents the change in enthalpy, ΔS the change in entropy, and R the universal gas constant [40].

(10) $$\ln x=\frac{∆H}{R}\frac{1}{T}-\frac{∆S}{R}$$

**Figure 11:**
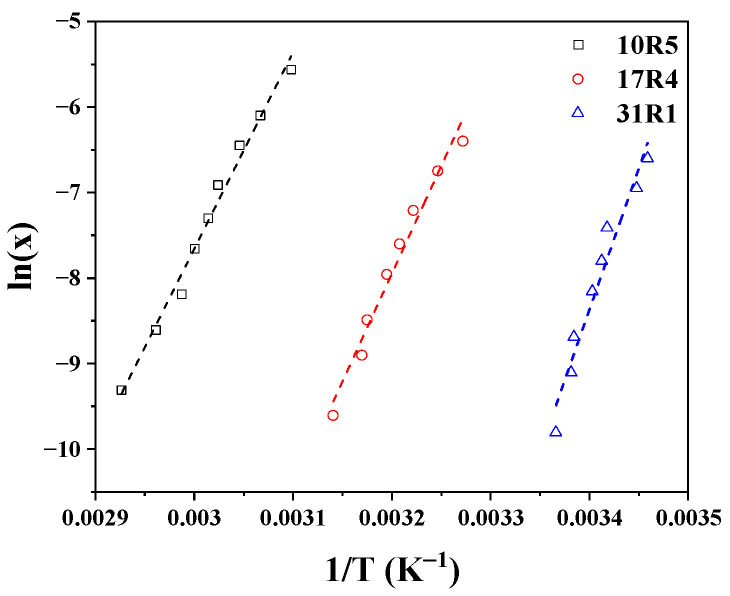
Natural logarithm of the mol fraction of copolymer as a function of the inverse of temperature of zone I to zone II transition for systems 10R5 (─□─), 17R4 (─○─), 31R1 (─△─).

Figure 11 shows the logarithm of x in molar fraction as a function of the reciprocal of the temperature, both corresponding to the transition from zone I to zone II for the three systems, whose behavior was fitted to straight lines. The fitting parameters, i.e., the slope and intercept, represent ΔH/R and ΔS/R, respectively.

The changes in Gibbs free energy can be calculated using the determined points and then adjusted using ΔG = ΔH − T ΔS. This is shown in Figure S14 of the Supplementary Materials. It can be seen that the profiles of ΔG decrease as the temperature increases. As the temperature increases, the proportion of hydrogen bonds in the copolymer decreases, while hydrophobic segment-segment interactions between the PPO blocks increase. This indicates disruption to the interactions between the PPO blocks and water, favoring the aggregation process. The interaction between polymeric systems of this type has been addressed in detail by Stetsyshyn Y. et al. [64].

A summary of the changes in enthalpy, entropy, and Gibbs free energy in the dehydration of PPO blocks are tabulated in Table 3.

One of the assumptions of Equation (10) is that the aggregation number in the structures remains constant within the studied concentration and temperature range, making it difficult to analyze this type of result due to significant variations between different authors [65]. Nevertheless, several remarks can be drawn from this.

According to Alexandridis et al. [10], positive enthalpy changes indicate that PPO dehydration is an unfavorable endothermic process, and the change in Gibbs free energy is negative, suggesting the formation of stable structures. The fact that the ΔG of copolymer 31R1 becomes less negative means that the process is “less spontaneous” or that dehydration in this system is more complicated due to larger hydrophobic segments [21]. These results are consistent in magnitude with those reported by other authors. Alexandridis et al. [10] calculated these changes in state properties for 12 direct Pluronics, whose values are very similar.

Thus, this process is “entropically governed,” since it is clear that TΔS > ΔH. According to Bouchemal et al. [66], a positive change in entropy occurs when hydrogen bonds are broken and water molecules are expelled from the hydrophobic segments of the unimers during the aggregation process. The change in entropy becomes greater here with the increase in the PPO/PEO ratio, as dehydration becomes more abrupt with ratio’s increase.

The values of ΔG, ΔH, and ΔS obtained by Naskar et al. [18] for the 10R5 copolymer are very similar to our own results, with magnitudes of −22.0 kJ/mol, 193.0 kJ/mol, and 0.72 kJ/(mol K), respectively, at 61 °C. In contrast, the values reported by Zhou and Chu [21] and Huff et al. [22] for 17R4 differ by half. This is because these researchers considered the flower-like micelle zone in their analysis. If the analysis were performed in the same way in this work, it is clear that very similar values would be obtained, since our CMT values were also similar. However, there is ambiguity over which values should be used in the analysis. If we were to analyze only the flower-like micelle zone, we would be able to do so for the 17R4 system only, due to the low micellization capacity of the other two systems.

## 4. Conclusions

In this study, the phase behavior and rheological properties of three reverse Pluronics with varying PPO/PEO ratios in aqueous solution were investigated: 10R5 [(PPO)_8_–(PEO)_22_–(PPO)_8_], 17R4 [(PPO)_14_–(PEO)_24_ –(PPO)_14_] and 31R1 [(PPO)_26_–(PEO)_7_–(PPO)_26_]. A combination of analytical techniques was employed, including visual observation, densimetry, sound velocity measurements, viscosimetry, tensiometry, dynamic light scattering, and rheometry.

The impact of the PPO/PEO ratio on solubility and micellization behavior was observed in all experimental trials. As predicted, an increase in the PPO/PEO ratio resulted in a decrease in both dehydration temperatures: PPO (corresponding to micellization) and PEO (corresponding to phase separation). Among the systems that were studied, 10R5/water exhibited the highest transition temperatures, while 31R1/water showed transitions occurring at temperatures below room temperature.

For systems 10R5 and 17R4, a zone of random networks was identified at low concentrations, a zone of flower-like micelles at medium concentrations, and a zone of micellar networks of interconnected flowers at high concentrations. It is noteworthy that 17R4 exhibited the most remarkable propensity for forming flower-like micelles, a property that can be attributed to its balanced PEO/PPO composition. In contrast, the 31R1 system exhibited only two structural regimes: random networks at low to intermediate concentrations and micellar networks at higher concentrations. The inability of 31R1 to form stable flower-like micelles is attributed to the short length of its central hydrophilic (PEO) block, which limits its capacity for loop formation and stable micelle architecture.

The diagrams of the 10R5 and 17R4 systems in aqueous solutions have been extended over a wider concentration and temperature range than previously reported in the literature. This objective was achieved by implementing a range of novel characterization methodologies, thereby facilitating the acquisition of new insights into the behavior of these systems. Moreover, a phase diagram for the 31R1 copolymer is presented for the first time. These phase diagrams are helpful in the context of polymer self-assembly or thermoresponsive material design.

The morphology of flower-like micelles is distinct, and consequently, their diffusion coefficients differ from those of other conformations. While DP are currently under extensive investigation for their potential roles in drug delivery and encapsulation, it is also worthwhile to assess the potential of flower-like micelles in these areas. That said, these micelles tend to form at concentrations that are relatively high for compatibility with the human body. Despite their low molecular weight, which could be advantageous, they are unlikely to be suitable for biomedical use on their own. Notwithstanding, their ability to thoroughly homogenize within a few days and their minimal foaming behavior indicate that they offer greater stability than many other surfactants.

This work presents a thorough analysis that builds on current knowledge of RP and encourages the exploration of new uses for these surfactants. Their notable stability, low foaming tendency, and strong wetting ability, reflected in their capacity to lower water’s surface tension to approximately 30 mN/m, make them promising candidates for alternative, non-biomedical applications.

## Figures and Tables

**Table 1:** Reverse Pluronics aggregate characteristics.

Structure	Characteristics	System Analyzed
Random networks	Visually cloudy dispersions. High polydispersity. Formed at low concentrations (1–10 wt.%).	10R5 [18,19], 17R4 [21,22], 25R8 [25].
Flower-like micelles	Visually transparent dispersions. Formed at medium concentrations (10–30 wt.%). Low polydispersity.	17R4 [21,22].
Micellar networks	Formed at high concentrations (>30% wt.%). Visually clear homogeneous dispersions. Predominantly viscous.	10R5 [19], 25R4 [24], 25R8 [25].
Lamellar Phase	Only high molecular weight reverse copolymers exhibit this phase. They form at high concentrations (>50 wt.%).	25R4 [24], 25R8 [25].

**Table 2:** Intercept α, dehydration energies (ΔE_DH_) and R^2^.

System	Region	Exp (α)	ΔE_DH_ (kJ/mol)	R^2^
10R5/water	I	0.406 ± 0.008	13.6 ± 0.02	0.9999
	II	0.423 ± 0.031	13.47 ± 0.08	0.9996
	III	5.075 × 10^−4^	32.36 ± 0.95	0.9965
17R4/water	I	0.833 ± 0.014	12.55 ± 0.03	0.9999
	II	0.610 ± 0.014	13.31 ± 0.04	0.9999
	III	2.359 × 10^−17^	113.64 ± 0.84	0.9988
31R1/water	I	1.027 ± 0.045	12.49 ± 0.12	0.9997
	II	1.518 ± 0.027	11.57 ± 0.06	0.9997
	III	1.137 × 10^−67^	398.15 ± 50.9	0.9683

**Table 3:** Changes in the state properties of the PPO dehydration process.

System	ΔH (kJ/mol)	ΔS (kJ/mol K)	T ΔS (kJ/mol)	ΔG (kJ/mol)
10R5/water	191.22 ± 8.18	0.6373 ± 0.0247	212.32 (60 °C)	−21.15 ± 0.08 (60 °C)
17R4/water	209.70 ± 13.91	0.7371 ± 0.0446	230.82 (40 °C)	−21.07 ± 0.07 (40 °C)
31R1/water	274.71 ± 24.44	1.0035 ± 0.0833	292.17 (18 °C)	−17.46 ± 0.29 (18 °C)

## Data Availability

Data can be found in the figures from the manuscript.

## Supplementary Materials

The following supporting information can be downloaded at: https://www.mdpi.com/article/10.3390/polym17152061/s1, Figure S1: Visual tests of the three systems obtained by direct observation. The lines represent the temperature at which the color change is observed C = clear, T = turbid, B = bluish, G = gel, 2ϕ = 2 phases, Figure S2: Derivative of sound velocity versus temperature of the 10R5/water system from visual observations. Concentrations in weight %: (a) 1 (──), 5 (──), (b) 10 (──), and 20 (──), Figure S3: Derivative of sound velocity versus temperature of the 31R1/water system from visual observations. Concentrations in weight %: (a) 1 (──), 5 (──), (b) 10 (──), and 20 (──), Figure S4: Apparent molar volume as a function of temperature of (a) 10R5/water and (b) 31R1/water. Apparent molar adiabatic compressibility as a function of temperature of (c) 10R5/water and (d) 31R1/water. The measurements being made at the concentrations in weight percent of: 1 (□), 3 (○), 5 (△), 10 (▽), 15 (⬠), 20 (◇), 30 (◁), 40 (▷), 50 (☆), 60 (⎔), and 80 (✳), Figure S5: Hydration number as a function of temperature of (a) 10R5/water and (b) 31R1/water; measurements at the concentrations in weight % of: 1 (□), 3 (○), 5 (△), 10 (▽), 15 (⬠), 20 (◇), 30 (◁), 40 (▷), 50 (☆), 60 (⎔), and 80 (✳). Hydration number as a function of concentration of (c) 10R5/water and (d) 31R1/water. Figure S6: Semi-static surface tension as a function of temperature of (a) 10R5/water and (b) 31R1/water at concentrations in weight % of: 0 (──), 1 (─□─), 2 (─☆─), 3 (─○─), 5 (─△─), 7 (─✳─), 10 (─▽─), and 20 (─◇─), Figure S7: Semi-static surface tension as a function of concentration and its linear fit, Figure S8: Increasing and decreasing temperature sweep for 10R5/water at concentrations of: (a) 15 and (b) 20 wt.%. 17R4/water at concentrations of: (c) 15 and (d) 20 wt. %, Figure S9: Viscosity as a function of reciprocal of temperature: (a) 10R5/water, (b) 17R4/water y (c) 31R1/water. Measurements at concentrations in weight % of: 0 (──), 1 (□), 3 (○), 5 (△), 10 (▽), 15 (⬠), 20 (◇), 30 (◁), 40 (▷), 50 (☆), 60 (⎔), and 80 (✳), Figure S10: Rheometry of the 10R5/water system, (a) moduli G′ and G″ as a function of frequency at 40% strain, (b) dynamic and shear viscosity (rheometry and viscometry) as a function of frequency and shear rate, (c) tan(δ) as a function of temperature, Figure S11: Rheometry of the 31R1/water system, (a) moduli G′ and G″ as a function of frequency at 40% strain, (b) dynamic and shear viscosity (rheometry and viscometry) as a function of frequency and shear rate, (c) tan(δ) as a function of temperature. Figure S12: Example of analysis by concentration of the 10R5/water system: (a) 20°C and (b) 40°C. Property measurements: density (─□─), sound velocity (─○─), molar volume (─△─), and viscosity (─▽─), Figure S13: Example of analysis by concentration of the 31R1/water system: (a) 6°C and (b) 15°C. Property measurements: density (─□─), sound velocity (─○─), molar volume (─△─), and viscosity (─▽─), Figure S14. Change in Gibbs free energy as a function of temperature for systems 10R5 (─□─), 17R4 (─○─), and 31R1 (─△─).
