# Supplementary material for: Effect of PPO/PEO Ratio on the Phase Behavior of Reverse Pluronics
Source: Polymers (Basel). 2025 Jul 28;17(15):2061. doi: 10.3390/polym17152061 (PMC12349445; doi:10.3390/polym17152061)
Supplement: Supplementary file 1 [file polymers-17-02061-s001.zip › polymers-3776738-supplementary.pdf]

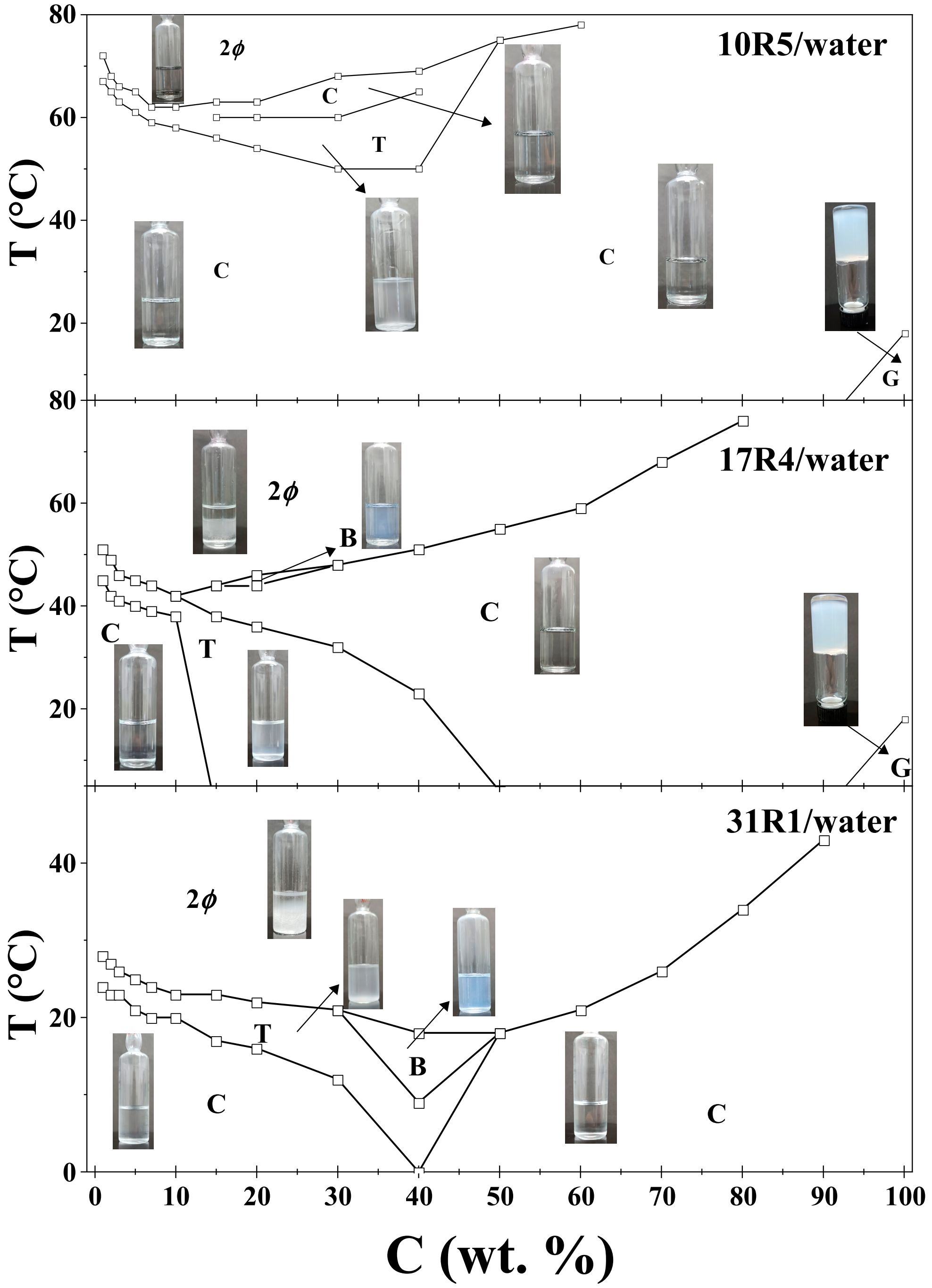

Figure S1. *Visual tests of the three systems obtained by direct observation. The lines represent the temperature at which the color change is observed.*
C = clear, T = turbid, B = bluish, G = gel, 2ϕ = 2 phases.

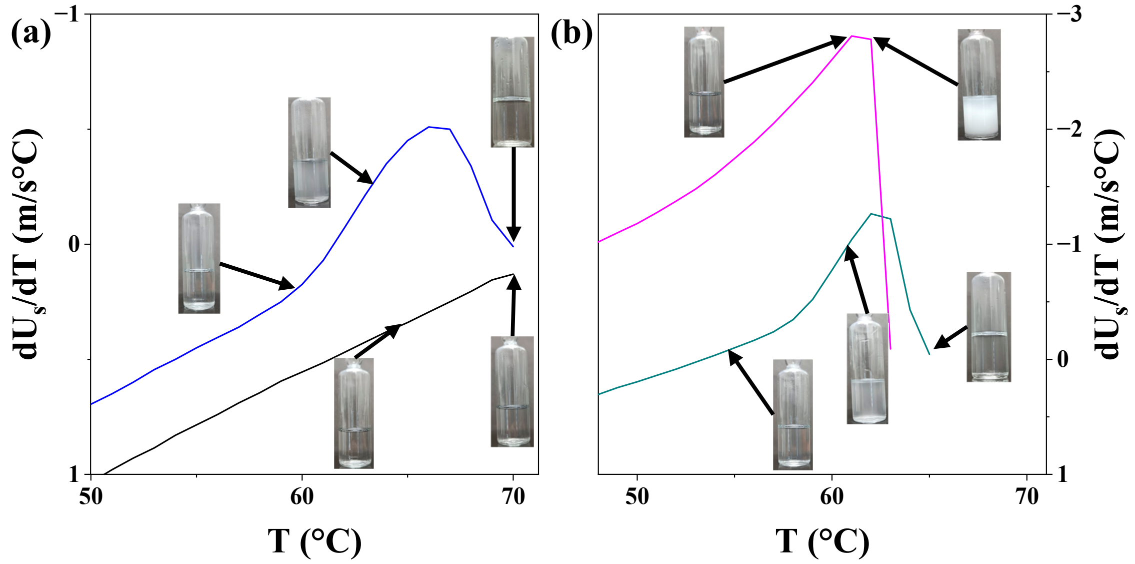

Figure S2. Derivative of sound velocity versus temperature of the 10R5/water system from visual observations. Concentrations in weight %: (a) 1 (—), 5 (—), (b) 10 (—), and 20 (—).

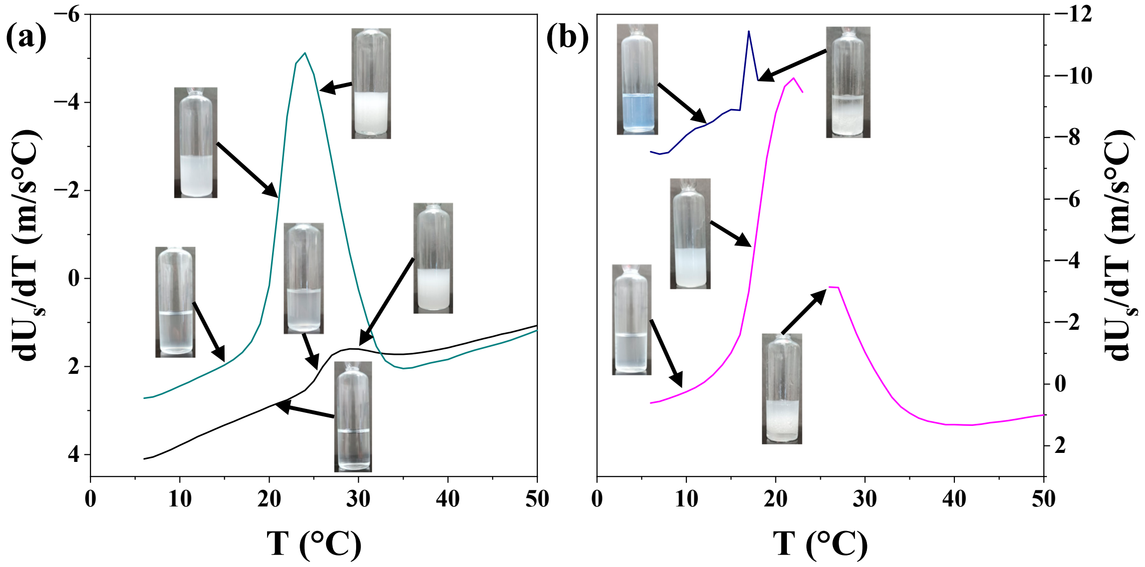

Figure S3. Derivative of sound velocity versus temperature of the 31R1/water system from visual observations. Concentrations in weight %: (a) 1 (—), 5 (—), (b) 10 (—), and 20 (—).

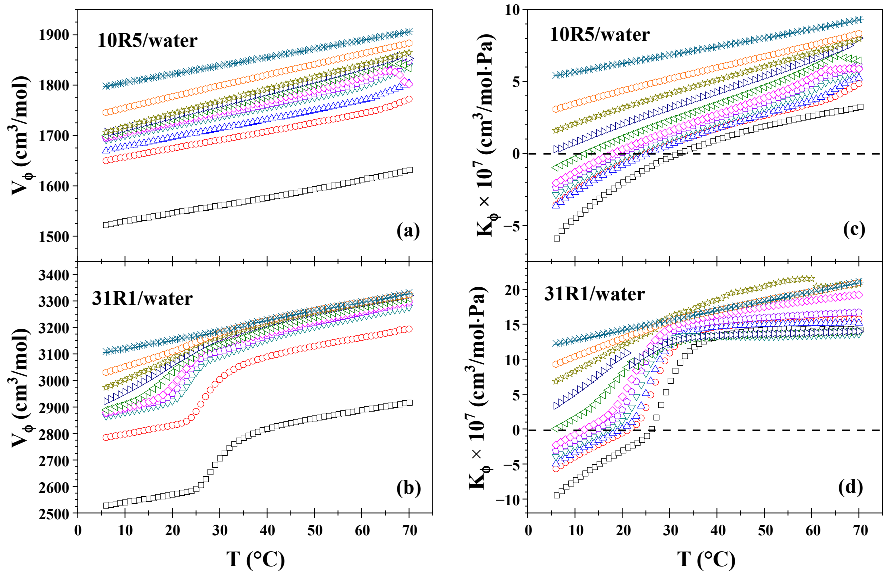

Figure S4. Apparent molar volume as a function of temperature of (a)10R5/water and (b) 31R1/water. Apparent molar adiabatic compressibility as a function of temperature of (c)10R5/water and (d) 31R1/water. The measurements being made at the concentrations in weight percent of: 1 (□), 3 (○), 5 (△), 10 (▽), 15 (⬠), 20 (◇), 30 (◁), 40 (▷), 50 (☆), 60 (⬡), and 80 (✳).

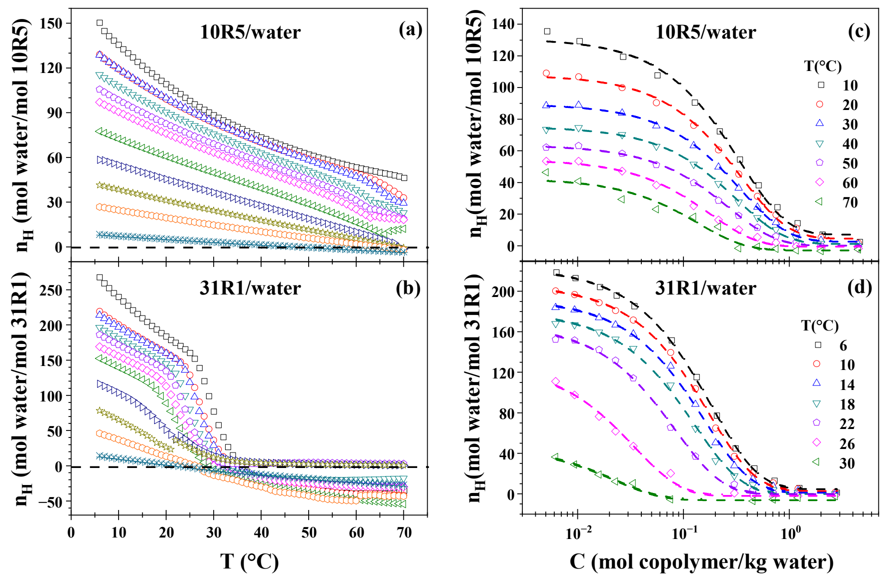

Figure S5. Hydration number as a function of temperature of (a)10R5/water and (b) 31R1/water.; measurements at the concentrations in weight % of: 1 (□), 3 (○), 5 (△), 10 (▽), 15 (⬠), 20 (◇), 30 (◁), 40 (▷), 50 (☆), 60 (⬡), and 80 (✳). Hydration number as a function of concentration of (c)10R5/water and (d) 31R1/water.

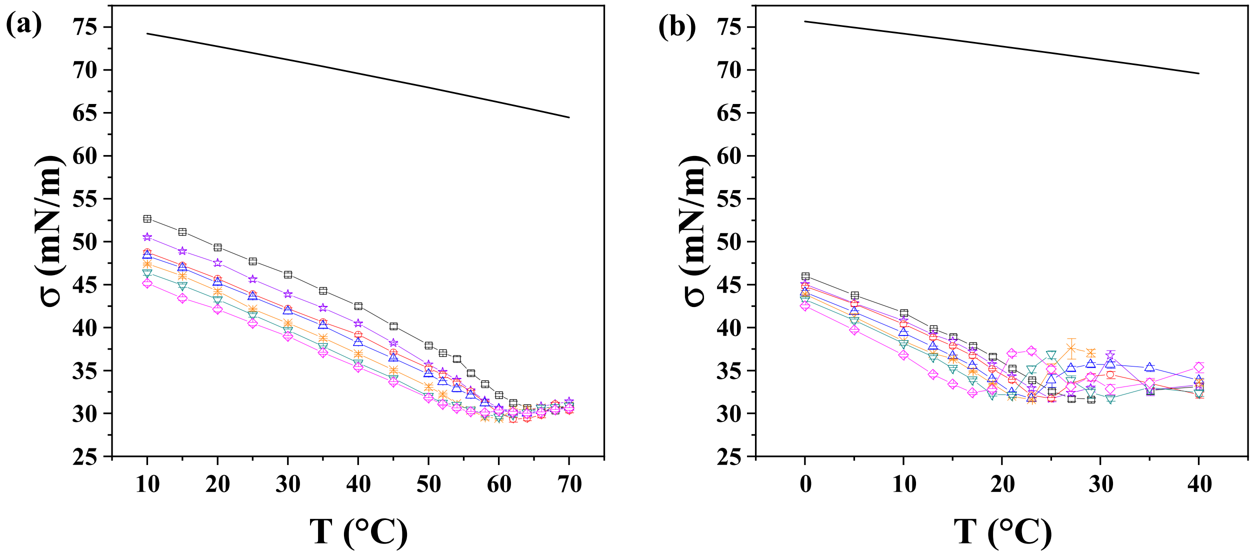

Figure S6. Semi-static surface tension as a function of temperature of (a)10R5/water and (b) 31R1/water at concentrations in weight % of: 0 (—), 1 (—□—), 2 (—☆—), 3 (—○—), 5 (—△—), 7 (—✳—), 10 (—▽—), and 20 (—◇—);

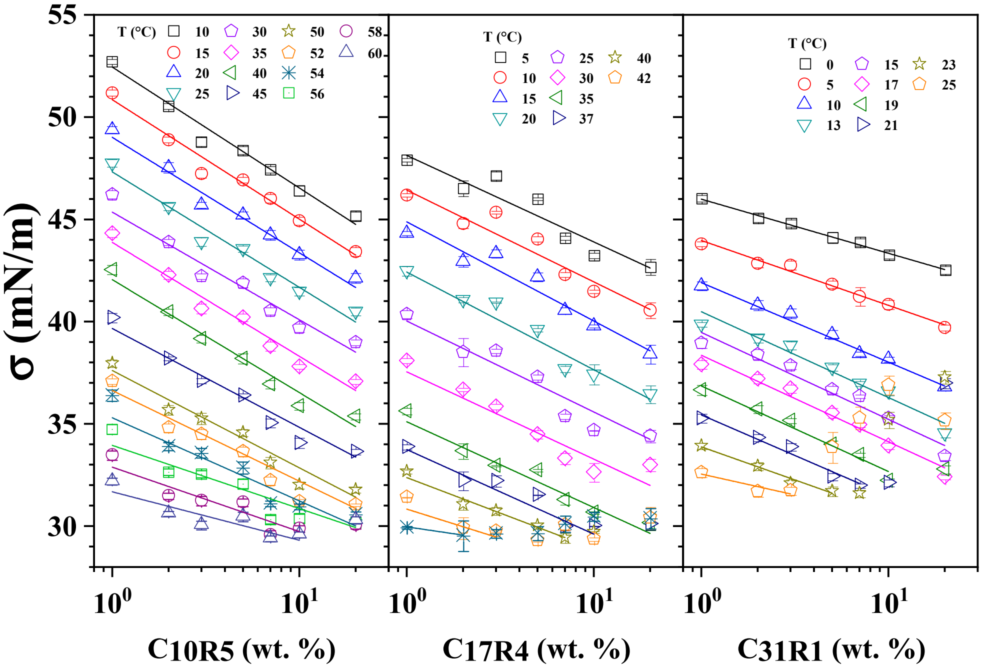

Figure S7. Semi-static surface tension as a function of concentration and its linear fit.

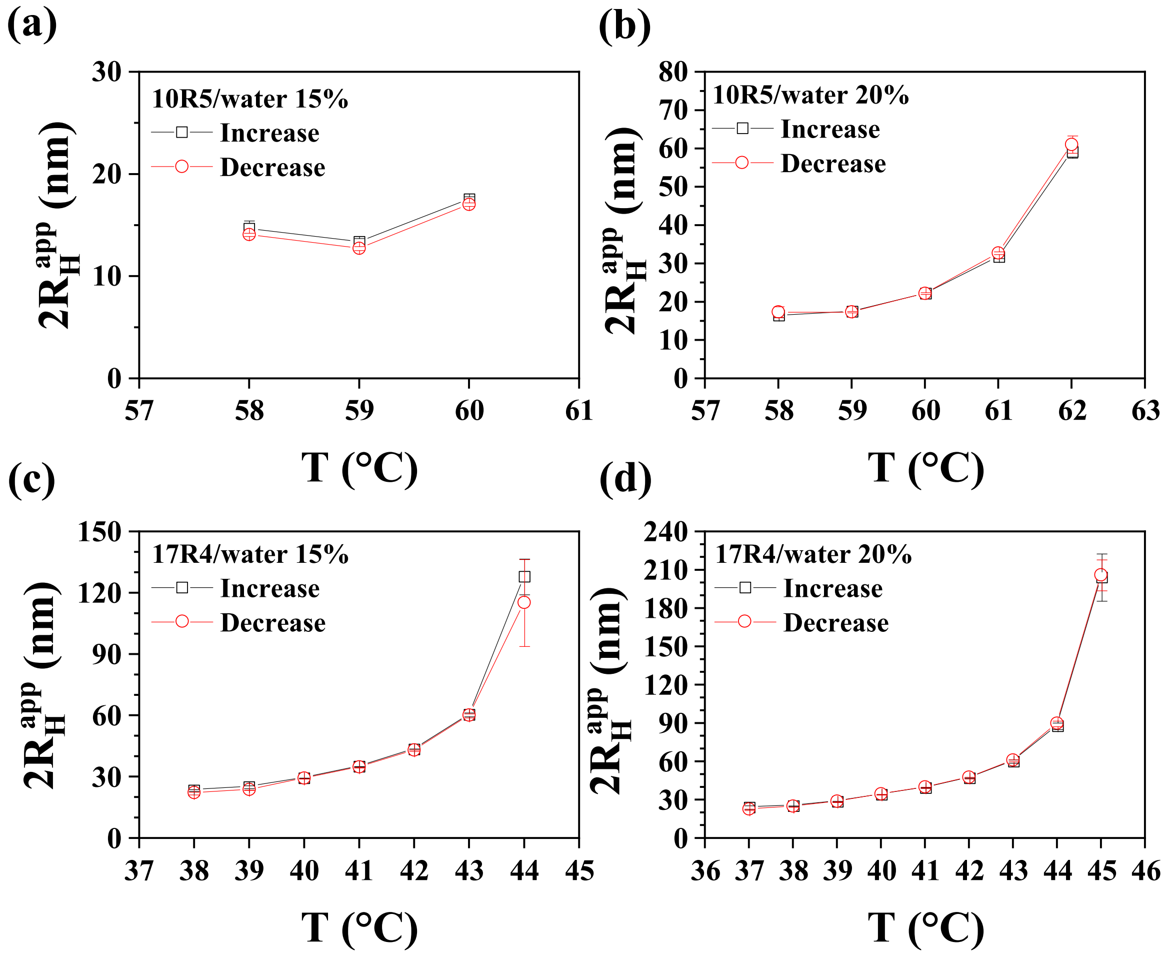

Figure S8. Increasing and decreasing temperature sweep for 10R5/water at concentrations of: (a) 15 and (b) 20 wt.%. 17R4/water at concentrations of: (c) 15 and (d) 20 wt. %.

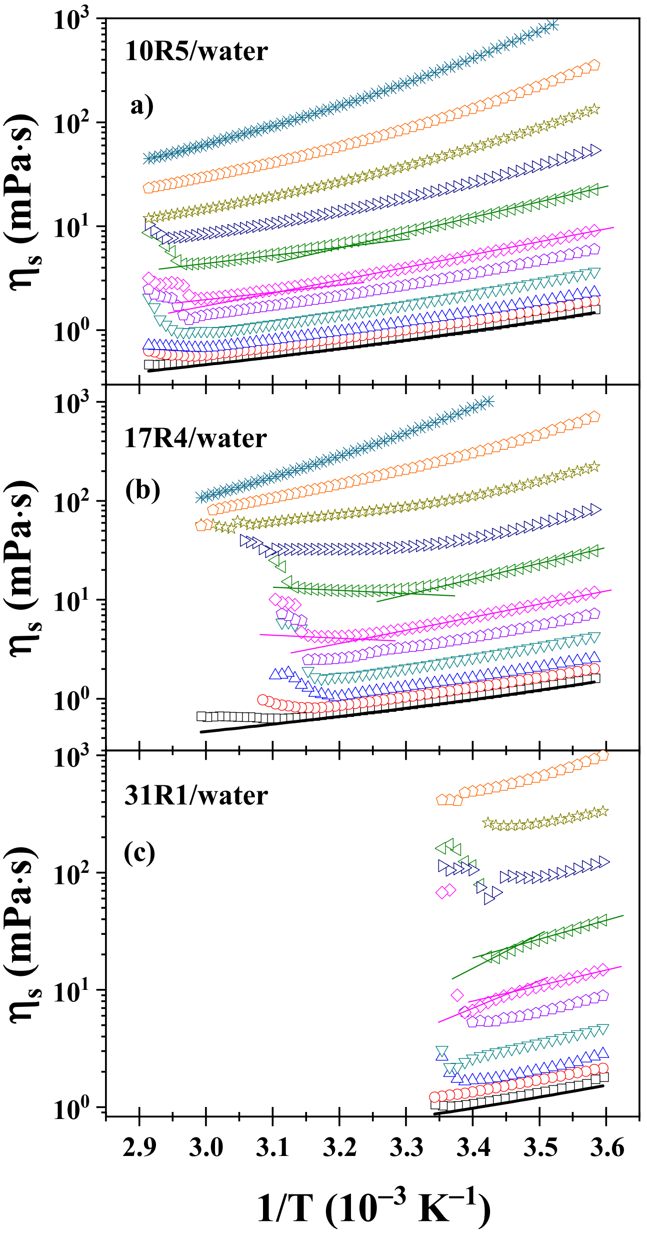

Figure S9. Viscosity as a function of temperature of: (a) 10R5/water, (b)17R4/water y (c) 31R1/water at concentrations in weight % of: 0 (—), 1 (□), 3 (○), 5 (△), 10 (▽), 15 (⬠), 20 (◇), 30 (◁), 40 (▷), 50 (☆), 60 (⬡), and 80 (✳).

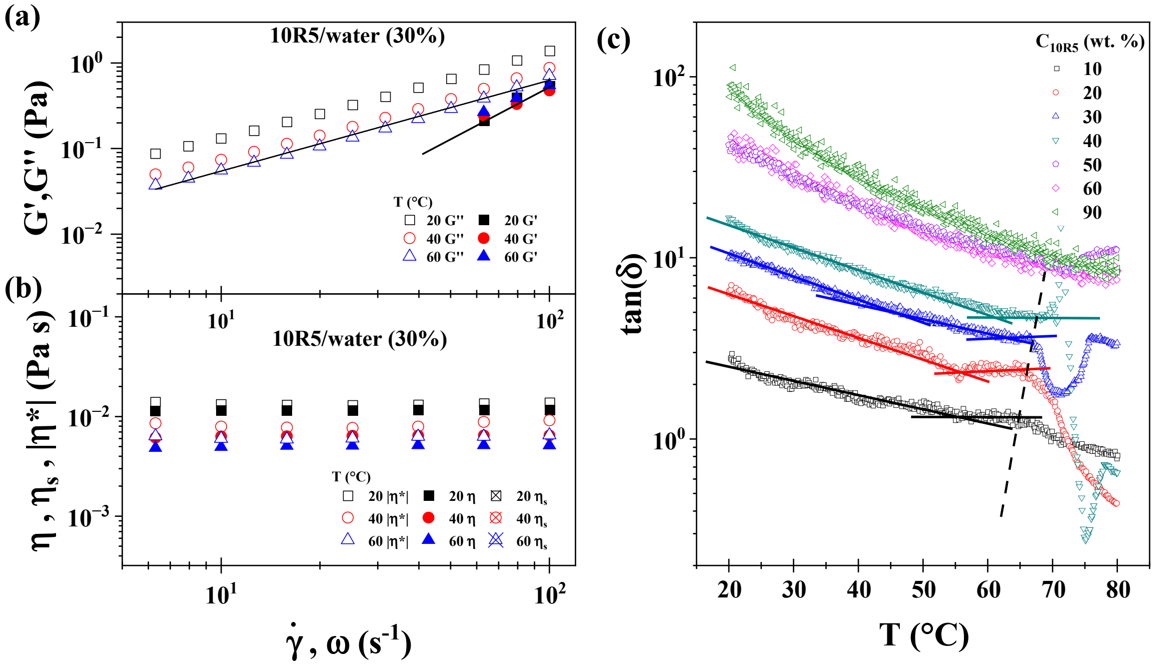

Figure S10. Rheometry of the 10R5/water system, (a) moduli G' and G'' as a function of frequency at 40% strain, (b) dynamic and shear viscosity (rheometry and viscometry) as a function of frequency and shear rate, (c) tan(δ) as a function of temperature.

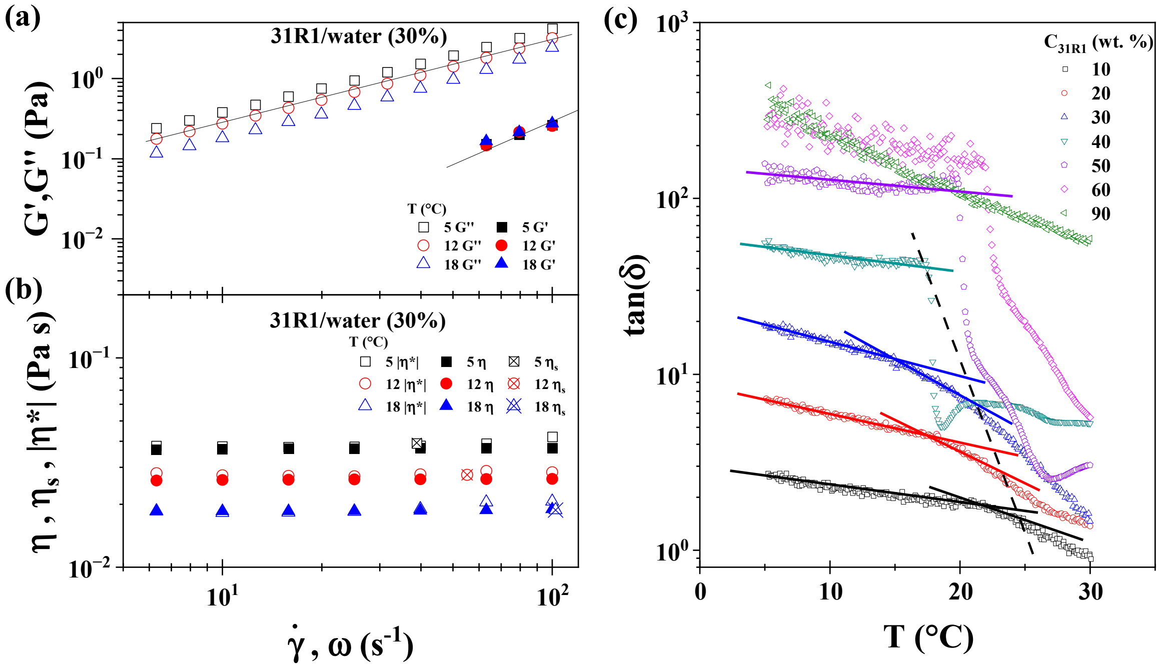

Figure S11. Rheometry of the 31R1/water system, (a) moduli G' and G'' as a function of frequency at 40% strain, (b) dynamic and shear viscosity (rheometry and viscometry) as a function of frequency and shear rate, (c) tan(δ) as a function of temperature.

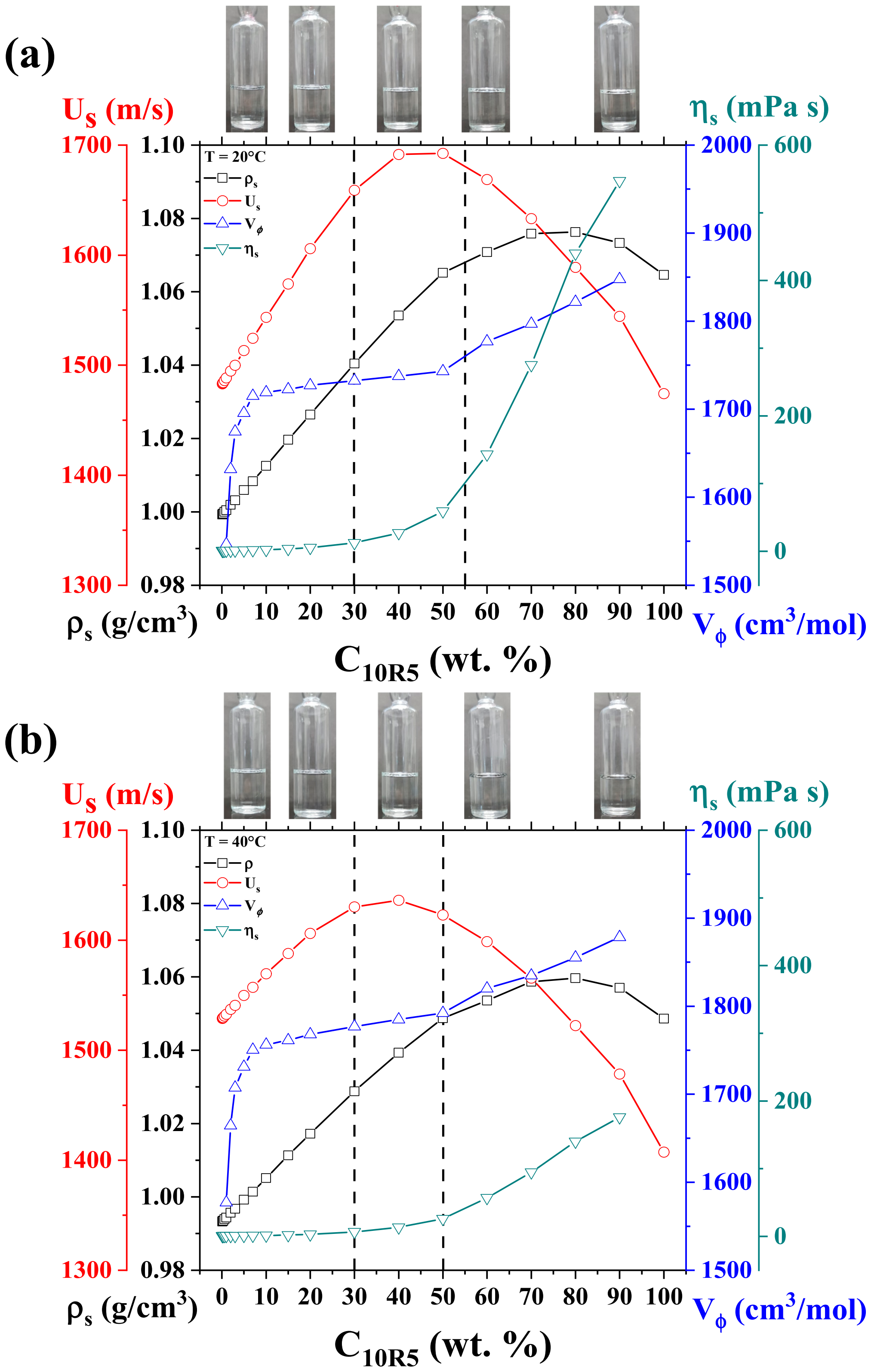

Figure S12. Example of analysis by concentration of the 10R5/water system: (a) 20°C and (b) 40°C. Property measurements: density (–□–), sound velocity (–○–), molar volume (–△–), and viscosity (–▽–).

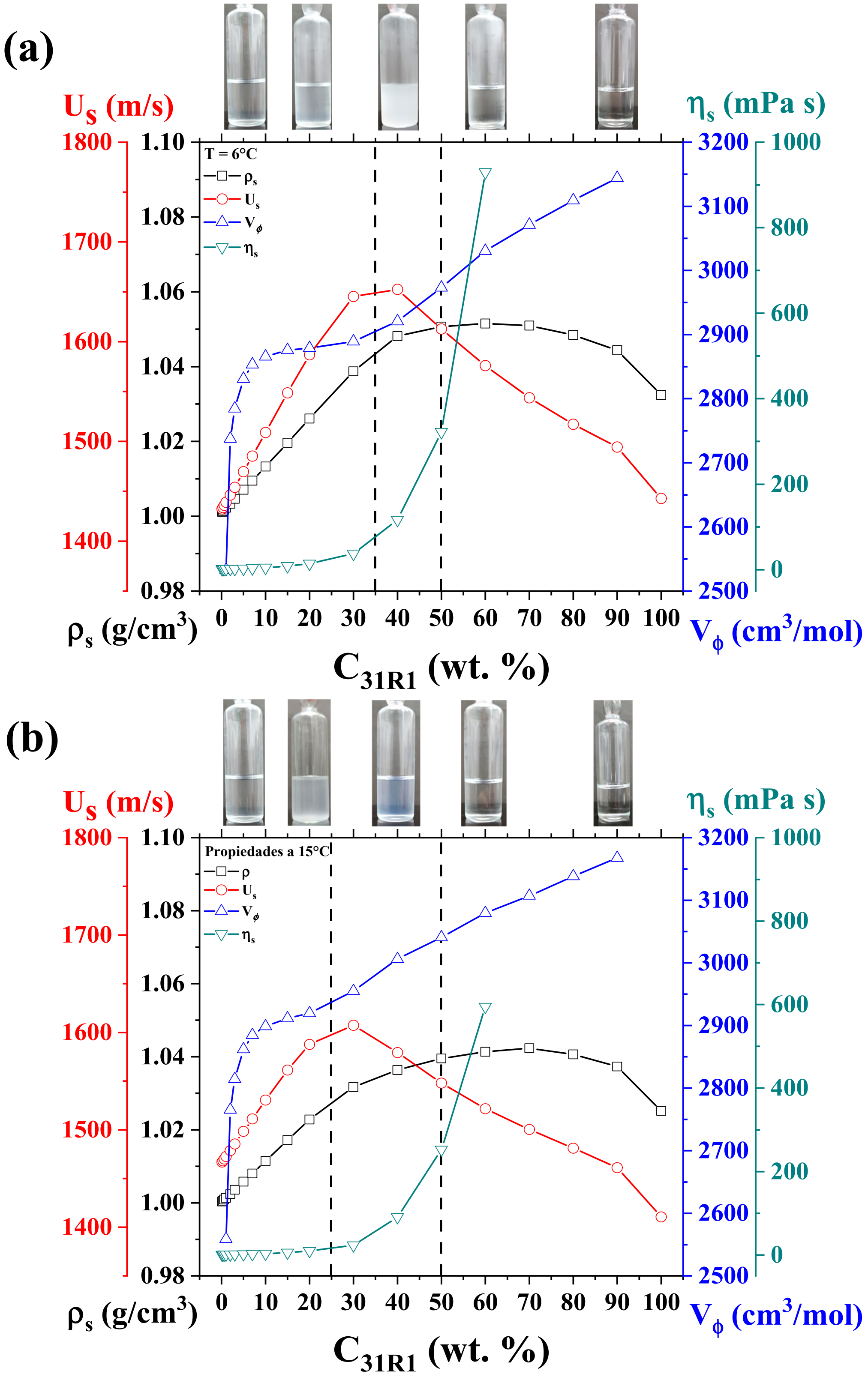

Figure S13. Example of analysis by concentration of the 31R1/water system: (a) 6°C and (b) 15°C. Property measurements: density (–□–), sound velocity (–○–), molar volume (–△–), and viscosity (–▽–).

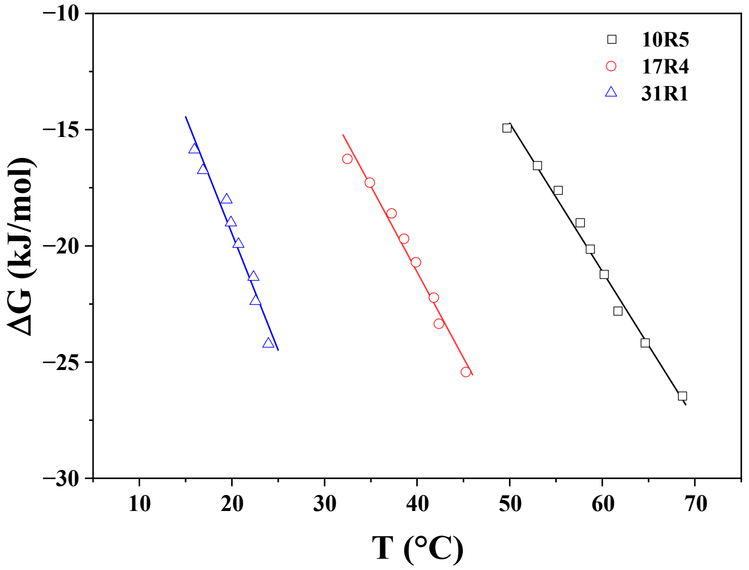

Figure S14. Change in Gibbs free energy as a function of temperature for systems 10R5 (–□–), 17R4 (–○–), and 31R1 (–△–).
